# The Many Layers of Membrane Biophysics: Environment-Sensitive Fluorophores Report on Structural Organization of Biological Membranes at Various Depths

**DOI:** 10.1021/acs.analchem.5c07236

**Published:** 2026-06-08

**Authors:** Florina Zakany, Rosemary Chandrakanthi Kothalawala, Olivér Pavela, Lajos Szente, Zoltan Varga, Gyorgy Panyi, Tamás Beke-Somfai, Peter Nagy, Tamas Kovacs

**Affiliations:** † Department of Biophysics and Cell Biology, Faculty of Medicine, 37599 University of Debrecen and MTA Centre of Excellence, Hungarian Academy of Sciences, Egyetem tér 1, Debrecen H-4032, Hungary; ‡ Biomolecular Self-assembly Research Group, Institute of Materials and Environmental Chemistry, HUN-REN Research Centre for Natural Sciences, Magyar Tudósok Körútja 2, Budapest H-1117, Hungary; § Hevesy György PhD School of Chemistry, Eötvös Loránd University, Pázmány Péter Sétány 1/A, Budapest H-1117, Hungary; ∥ 368105 CycloLab Cyclodextrin R&D Laboratory Ltd., Illatos u. 7., Budapest H-1097, Hungary

## Abstract

Molecular-order-related membrane biophysical properties contribute to the functional modulation of transmembrane proteins, and therefore, their changes due to a modified membrane composition in diseases associated with alterations in lipid levels may play important pathophysiological roles. In living cells, membrane biophysics can be examined with environment-sensitive fluorophores that describe the structural organization of biological bilayers from different aspects. Microviscosity-sensitive probes such as TMA-DPH characterize the degree of motional freedom, that is, fluidity, polarity-sensitive dyes including Laurdan or PY3174 report on the extent of water penetration, that is, hydration, whereas voltage-sensitive fluorophores such as di-8-ANEPPS quantify the nonrandom alignment of molecular dipoles, that is, the dipole potential. Given that all of the above properties are intrinsically linked to the arrangement of membrane constituents, such parameters are assumed to change in parallel with each other, and thus, the information gained by the environment-sensitive fluorophores is generally considered equivalent. In the current study, using fluorescence-based measurement techniques and manipulating membrane sterol levels with cyclodextrin-based complexes in living cells, we experimentally demonstrate incongruent changes in fluorescence properties of TMA-DPH, Laurdan, PY3174, and di-8-ANEPPS. Comparative MD simulations reveal that this can be due to the distinct membrane localization of the fluorophores. Our experimental and computational analyses reveal that the most commonly applied environment-sensitive fluorophores depict the structural organization of membranes at different depths and suggest that a comprehensive investigation of bilayer structure should include an appropriate combination of dyes, which would be required for a better understanding of membrane biophysics and its potential roles in disease pathogenesis.

## Graphical abstract

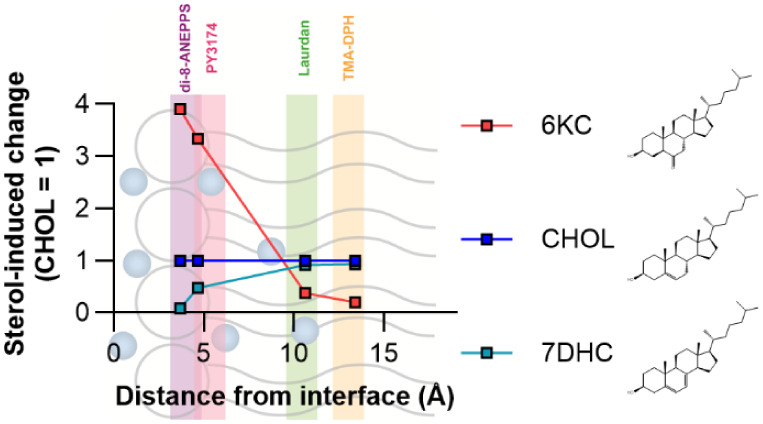

Owing to the embedding of their transmembrane domains into biological lipid bilayers, transmembrane proteins, such as ion channels, ABC transporters, G protein-coupled receptors, or receptor tyrosine kinases, are susceptible to substantial modulatory interactions with membrane lipids, which arise through a mixture of direct, ligand-like lipid–protein interactions and indirect effects mediated by bulk biophysical membrane properties.^1,2^ While direct lipid effects are characterized relatively well due to the recent development of computational structural techniques such as X-ray crystallography, cryo-electron microscopy, and molecular dynamics simulations, much less is known about indirect modulation by lipids, which can be at least partially related to methodological limitations. Indirect lipid actions are linked to the structural organization of membrane constituents that, in living cells, are generally described by three parameters, membrane fluidity, hydration, and dipole potential. While all of the three are intrinsically related to the arrangement of membrane constituents, they characterize membrane structure from different aspects. Namely, fluidity refers to the motional, mainly rotational freedom of molecules; hydration is the extent of water penetration into the bilayer; and dipole potential is a large positive intramembrane potential generated by the nonrandom orientation of molecular dipoles at the membrane–water interface.^3–7^ The latter two represent partially overlapping features of the membrane considering that anisotropically ordered water molecules at the membrane–water interface substantially determine the magnitude of dipole potential and also contribute to the superficial hydration of the membrane. However, the definition of membrane hydration includes all water molecules within the membrane whereas the dipole potential is associated mainly with the interfacial ones. Furthermore, while ordered water molecules determine dipole potential to a large extent, other factors contribute to its magnitude such as carbonyl groups of the ester linkages between the headgroup and the hydrocarbon chains or the P^–^-N^+^ dipole of the lipid headgroup of phospholipids, and the amount of sterols as well, which justifies the differentiation between the two related properties.^3–7^

In living cells, these parameters are examined by environment-sensitive fluorophores and fluorescence-based measurement techniques. Membrane fluidity can be tested with TMA-DPH (4′-(trimethylammonio)-diphenylhexatriene) since its fluorescence anisotropy negatively correlates with the rotational freedom of the fluorophore.^8–13^ Membrane hydration can be quantified using Laurdan (6-dodecanoyl-*N*,*N*-dimethyl-2-naphthylamine) since its emission spectrum is changed as a function of the hydrophobicity of its microenvironment and, therefore, the generalized polarization quantifying its spectral shift negatively correlates with the degree of water penetration into bilayers.^8–11,14–18^ Due to the unfavorable spectral properties of Laurdan with its excitability falling mainly in the UV range, several functional analogue compounds, for example, PY3174 (4-[2-(6-dibutylamino-5-fluoro-naphthalen-2-yl)-vinyl]-1-(3-triethylammonio-propyl)-pyridinium dibromide), were proposed as substitutes.^11,19,20^ The magnitude of dipole potential can be estimated with voltage-sensitive dyes such as di-8-ANEPPS (4-(2-[6-(dioctylamino)-2-naphthalenyl]­ethenyl)-1-(3-sulfopropylpyridinium inner salt) that displays shifts to lower wavelengths in its excitation spectrum in response to larger magnitudes of the surrounding, intramembrane electric field and can thus be utilized in an excitation ratiometric assay to estimate the value of the dipole potential.^8,10,20–29^

The biophysical properties of biological membranes are essentially determined by their lipid composition. Although certain factors such as the type, acyl chain length, and saturation of phospholipids,^20,29–31^ as well as the abundance of glucosylceramides and ceramides, also influence these parameters,^9,10,13,18,26,32^ they are fundamentally determined by the amount of cholesterol in the bilayer. Namely, a higher membrane cholesterol level reduces membrane fluidity^11,12,20,30,33–35^ and hydration^11,14–17,20^ and increases the magnitude of dipole potential.^8,20,22–24,26,27,36–38^ Other sterols also alter membrane biophysical properties. For example, 7-dehydrocholesterol reduces fluidity and hydration as well^20,39^ while elevating dipole potential much less effectively than cholesterol.^20,24,28^ On the other hand, 6-ketocholestanol very efficiently increases dipole potential^8,20,21,23,25^ without largely influencing fluidity or hydration.^8,20^ The amounts of sterols in biological membranes can be efficiently altered by cyclodextrins composed of seven α-1,4-d-glucopyranoside units (βCDs) forming a toroidal, hollow, truncated cone-shaped structure with a hydrophobic interior and a hydrophilic outer surface.^3,40,41^ βCDs are typically utilized to form water-soluble noncovalent host–guest inclusion complexes with otherwise poorly soluble hydrophobic substances such as various drugs and sterols.^40,42^ In accordance, empty βCDs, particularly the methylated derivative (MβCD), efficiently extract cholesterol molecules from biological membranes and are thus widely applied for membrane cholesterol depletion in cellular studies.^3,43,44^ On the other hand, when precomplexed, MβCD can efficiently load the cell membrane with sterols as it was previously demonstrated for cholesterol, 7-dehydrocholesterol, and 6-ketocholestanol as well.^20,28,44–46^

Due to the fact that membrane fluidity, hydration, and dipole potential are intrinsically order-related properties of bilayers, they are generally assumed to change in parallel with each other in response to alterations in membrane lipid levels, and accordingly, they are often collectively and somewhat dismissively referred to as “membrane order” or “lipid order” in the literature. Nevertheless, their definition may also imply that these parameters characterize molecular organization at various depths of bilayers. Namely, although these properties can overlap, fluidity may particularly characterize the interior hydrophobic core, dipole potential at the membrane–water interface, whereas hydration characterizes the intermediate regions between the two. However, their interrelationship is not experimentally proven in living cells and the nomenclature of these parameters remains thus rather incoherent in the literature. In accordance, while fluorescence parameters of environment-sensitive dyes are generally thought to shift in parallel with each other, their thorough comparative analysis has not been performed yet. Therefore, in this study, we apply various environment-sensitive fluorophores including TMA-DPH, Laurdan, PY3174, and di-8-ANEPPS, and sterol-MβCD complexes to study the effects of cholesterol, 7-dehydrocholesterol, and 6-ketocholestanol on the membrane biophysical properties of cells. We demonstrate that these sterols distinctively alter membrane biophysics since while cholesterol reduces both membrane fluidity and hydration and elevates the magnitude of dipole potential, 7-dehydrocholesterol is only able to efficiently alter fluidity and hydration, and 6-ketocholestanol only increases notably the dipole potential. We also show that sterol-induced alterations in PY3174 generalized polarization, in spite of the dye being initially proposed as a Laurdan alternative, more closely resemble that of the di-8-ANEPPS excitation ratio. Furthermore, by applying MD simulations in a simplified bilayer environment, we provide evidence that these fluorophores characterize the membrane structure at various depths as TMA-DPH and Laurdan report on molecular organization at deeper, more hydrophobic layers, whereas PY3174 and di-8-ANEPPS report rather at the membrane–water interface. Our results demonstrate that membrane biophysical properties can be properly characterized by a combination of environment-sensitive fluorophores that report on the structural organization at different depths of bilayers, and MβCD complexes of sterols efficiently and distinctively alter these parameters, which may be invaluable tools to examine membrane biophysics-related changes in protein function.

## Materials and Methods

### Cell Culture and Treatments

The Chinese hamster ovary (CHO) cell line was obtained from the American Type Culture Collection (Manassas, VA) and grown according to its specifications. For spectrofluorometry measurements, cells were harvested at a confluence of 80–90% by trypsinization, while for confocal microscopy, cells were grown on 8-well chambered coverglass (ibidi, Gräfelfing, Germany).

To manipulate sterol levels of the cell membrane, cells were loaded with 6-ketocholestanol (3β-hydroxy-5α-cholestan-6-one, 6KC) (Sigma-Aldrich, St. Louis, MO), cholesterol (CHOL) (Sigma-Aldrich), or 7-dehydrocholesterol (7DHC) (Sigma-Aldrich) using custom-synthesized sterol-methyl-beta-cyclodextrin (MβCD) complexes (CycloLab Cyclodextrin R&D Laboratory, Budapest, Hungary) at sterol concentrations of 200 μM for 60 min at room temperature. As controls, cells were treated with the corresponding amounts of native MβCD complexes (CycloLab Cyclodextrin R&D Laboratory) not containing sterols. Native and sterol-loaded complexes were dissolved, and treatments of cells were carried out in normal Ringer’s solution.

### Measurement of the Fluorescence Anisotropy of TMA-DPH

To quantify the fluorescence anisotropy of 4′-(trimethylammonio)-diphenylhexatriene (TMA-DPH) (Sigma-Aldrich) as described previously,^8–11,20^ trypsinized untreated CHO cells and those treated with native or sterol-loaded MβCD complexes for 1 h were labeled with 10 μM TMA-DPH in Hank’s buffer for 20 min at room temperature. Labeled cells were diluted in Hank’s buffer without washing to a concentration of 10^6^/ml, and fluorescence intensities were measured with a Fluorolog-3 spectrofluorometer (Horiba Jobin Yvon, Edison, NJ) with the temperature of the cuvette holder adjusted to 37 °C by a circulating water bath.

The fluorescence anisotropy (*r*) of TMA-DPH was determined in the L-format after an excitation at 352 nm and measurement of fluorescence intensities at 430 nm according to eq 1.

1 $$r=\frac{I_{vv}-GI_{vh}}{I_{vv}+2GI_{vh}}$$

In eq 1, *I*_vv_ and *I*_vh_ are the vertical and horizontal components, respectively, of the fluorescence after excitation by vertically polarized light, and *G* is an instrument-specific correction factor characterizing the different sensitivity of the detection system for vertically and horizontally polarized light calculated according to eq 2.

2 $$G=\frac{I_{hv}}{I_{hh}}$$

In eq 2, *I*_hv_ and *I*_hh_ are the vertical and horizontal components, respectively, of the fluorescence after excitation by horizontally polarized light.

### Determination of the Generalized Polarization of Laurdan

To determine the generalized polarization of 6-dodecanoyl-*N*,*N*-dimethyl-2-naphthylamine (Laurdan) (Sigma-Aldrich) using conventional confocal microscopy as in recent studies,^47–50^ cells were grown onto an 8-well chambered coverglass. Subsequently, untreated CHO cells and those treated with native or sterol-loaded MβCD complexes for 1 h were stained with 2 μM Laurdan in Hank’s buffer for 20 min at room temperature, and images were taken at the midplane of the cells using an LSM880 confocal laser-scanning microscope (Carl Zeiss AG, Jena, Germany) equipped with an enclosed chamber surrounding the entire microscope stage area, and the temperature was adjusted and maintained at 37 °C for the measurements. After a 405 nm excitation, spectral images were acquired using the lambda mode of the microscope with a Quasar 32-channel GaAsP detector array between 410 and 563 nm with a bandwidth of 8.9 nm. During processing, segmentation of images into membrane and nonmembrane pixels was carried out with the manually seeded watershed algorithm using a custom-written MATLAB (MathWorks, Natick, MA) program, which was followed by the identification of individual cells. The detected fluorescence intensities were integrated between 419 and 437 nm (*I*_blue_) and 473 and 500 nm (*I*_red_) and the median value of the generalized polarization (GP) of Laurdan was calculated from the data of cell membrane pixels for each individual cell after background subtraction according to eq 3.

3 $$GP=\frac{I_{\mathrm{blue}}-I_{\mathrm{red}}}{I_{\mathrm{blue}}+I_{\mathrm{red}}}$$

Alternatively, to examine generalized polarization with the most widely applied spectrofluorometry method as in our previous studies,^8,10,11^ trypsinized CHO cells treated and labeled as above were washed and resuspended in Hank’s buffer at a concentration of 10^6^/ml. Fluorescence intensities were subsequently measured with a Fluorolog-3 spectrofluorometer with the temperature of the cuvette holder adjusted to 37 °C by a circulating water bath.

The dye was excited at 350 nm, and its emission was detected in the blue range of its emission spectrum at 435 nm (*I*_blue_) and at the red edge at 500 nm (*I*_red_). Generalized polarization of Laurdan fluorescence was calculated according to eq 3.

### Quantification of the Generalized Polarization of PY3174

The generalized polarization of PY3174 (di-4-AN­(F)­EPPTEA; 4-[2-(6-dibutylamino-5-fluoro-naphthalen-2-yl)-vinyl]-1-(3-triethylammonio-propyl)-pyridinium dibromide) (Potentiometric Probes, Farmington, CT), synthesized and proposed to be a Laurdan analogue,^19^ was quantified using confocal microscopy as previously.^11,20^ For these measurements, cells were grown onto an 8-well chambered coverglass. Subsequently, untreated CHO cells and those treated with native or sterol-loaded MβCD complexes for 1 h were stained with 10 μM PY3174 for 20 min at room temperature and images were taken at the midplane of cells using an LSM880 confocal laser-scanning microscope equipped with an enclosed chamber surrounding the entire microscope stage area and the temperature was adjusted and maintained at 37 °C for the measurements. PY3174 was excited at 488 nm, and spectral images were acquired using the lambda mode of the microscope with a Quasar 32-channel GaAsP detector array between 500 and 698 nm with a bandwidth of 8.9 nm. During processing, segmentation and analysis of images were carried out as described above for Laurdan. Emitted intensities were integrated in two wavelength ranges between 509 and 572 nm (*I*_blue_) and 608 and 671 nm (*I*_red_), and the median value of the generalized polarization of PY3174 was calculated from the data of cell membrane pixels for each individual cell after background subtraction using eq 3.

Alternatively, to investigate the generalized polarization of PY3174 with spectrofluorometry, trypsinized CHO cells treated and labeled as above were examined with a Fluorolog-3 spectrofluorometer at 37 °C. The emission spectrum of PY3174 was recorded after a 488 nm excitation by varying the emission wavelength between 500 and 750 nm with an increment of 1 nm using slits adjusted to 5 nm on both the excitation and emission sides. The generalized polarization of PY3174 was calculated using eq 3 after integrating fluorescence intensities corresponding to *I*_blue_ and *I*_red_ ranges as in confocal microscopy.

### Measurement of the Excitation Ratio of Di-8-ANEPPS

To quantify alterations in the excitation ratio of the 4-(2-[6-(dioctylamino)-2-naphthalenyl]­ethenyl)-1-(3-sulfopropyl)­pyridinium inner salt (di-8-ANEPPS) (Thermo Fisher Scientific, Waltham, MA) voltage-sensitive fluorophore using confocal microscopy as previously,^10,25,26^ cells were grown onto an 8-well chambered coverglass. Untreated CHO cells and those treated with native or sterol-loaded MβCD complexes for 1 h were labeled with 2 μM di-8-ANEPPS for 20 min at room temperature, and images were subsequently taken at the midplane of cells using an LSM880 confocal laser-scanning microscope equipped with an enclosed chamber surrounding the entire microscope stage area, and the temperature was adjusted and maintained at 37 °C for the measurements. Di-8-ANEPPS was excited at 458 and 514 nm, and its emitted intensities were measured between 584 and 686 nm (*I*_blue_ and *I*_red_ after excitation at 458 and 514 nm, respectively). During quantitative image analysis, cell membrane pixels were identified for each individual cell as described above. The median value of the fluorescence intensity ratio (*R*_exc_ = *I*_blue_/*I*_red_) was subsequently calculated from the data of cell membrane pixels for each individual cell after background subtraction.

Alternatively, to investigate the excitation ratio of di-8-ANEPPS with spectrofluorometry, trypsinized CHO cells treated and labeled as above were examined with a Fluorolog-3 spectrofluorometer at 37 °C. The excitation spectrum of the dye was recorded by varying the excitation wavelength between 380 and 550 nm with an increment of 1 nm and measuring the emission at 660 nm using slits adjusted to 5 nm on both the excitation and emission sides. The excitation ratio was calculated as in confocal microscopy.

### MD Simulations

The structures of TMA-DPH, Laurdan, PY3174, and di-8-ANEPPS molecules were obtained from quantum mechanics (QM) optimized geometries, which were also used for electrostatic potential (ESP) calculations. QM calculations were performed at the B3LYP/6–31G*/HF/6–31G* level of theory. The atomic partial charges for the four fluorophores were derived with the restrained electrostatic potential (RESP) methodology^51^ (restraint weight of 0.01) from previous ESP calculations. GAFF force field parameters were used to describe the valence parameters for the fluorophores.^52^

The fluorophore molecules were embedded into a pre-equilibrated lipid bilayer composed of 1-palmitoyl-2-oleoyl-*sn*-glycero-3-phosphocholine (POPC) built with the CHARMM-GUI web tool.^53^ The initial orientation of the fluorophore molecules in the lipid bilayer had an angle of approximately 30° relative to the membrane normal. Model 1 for all four fluorophore molecules has the selected atoms (N1 in TMA-DPH, N1 in Laurdan, N2 in PY3174, N1 in di-8-ANEPPS) at the same depth to the phospholipid membrane surface, and in Model 2 and Model 3 of each fluorophore, the dyes were moved toward the center of the bilayer by 5 Å and 10 Å, respectively. Additionally, the system was solvated in a TIP3P^54^ bilayer water shell and neutralized by adding chloride ions. Finally, 150 mM NaCl was added to simulate physiological salt concentration. The valence parameters of POPC were adopted from Lipid14.^55^ A total of 12 fluorophore–bilayer complex systems were generated.

Energy minimization was performed on the entire system for 2000 steps, with the first 1000 steps utilizing the steepest descent method and the subsequent 1000 steps employing the conjugate gradient method, during which restraints were applied to the phospholipid head groups and ligand heavy atoms (100 kcal·mol^–1^·Å^–2^). Subsequently, the same optimization process was carried out, but with restraints applied solely to the ligand (100 kcal·mol^–1^·Å^–2^). Then, the entire system was gradually heated to 300 K over 40 ps under the same restraint conditions. To balance the system density, a 100 ps equilibration simulation was performed under the NPT ensemble at 300 K to balance the system density, using the CPU version of PMEMD from the AMBER22 software package^56^ with smaller restraints applied to the ligand heavy atoms (10 kcal·mol^–1^·Å^–2^). All restraints were removed in 1 ns equilibration simulations to further optimize the system density, performed with the GPU implementation of PMEMD. Finally, a 250 ns production MD simulation was performed since generally stable fluorophore positions were obtained within this time interval. However, in the case of PY3174, two simulations were extended to 400 ns due to a large-scale positional change, whereas, in one simulation, Laurdan underwent a flip in the orientation and thus the duration was also extended to 400 ns in that case. Atomic coordinates of all atoms were recorded every 10 ps. The temperature was controlled using the Langevin thermostat,^57^ while pressure was controlled using the anisotropic Berendsen barostat.^58^ Bond lengths involving hydrogen atoms were constrained using the SHAKE algorithm.^59^ van der Waals interactions were treated using a nonbonding cutoff of 10 Å, and electrostatic interactions were calculated with the particle mesh Ewald method.^60^

The membrane thickness was measured on the basis of the average distance between the P atoms in the upper and lower layers. The tilt angle to membrane normal was measured based on the selected atoms in each fluorophore molecule: N1 and C4 in TMA-DPH; N1 and C12 in Laurdan; N2 and N3 in PY3174; and N1 and N2 in di-8-ANEPPS (for the numbering of atoms, see Figure 5). The positional RMSD for each fluorophore molecule was calculated based on their heavy atoms. The two alkyl tails in di-8-ANEPPS were excluded in the calculations of positional RMSD due to their large flexibility. Distances to the interface were calculated based on the selected atoms in each fluorophore to the surface with the P atoms in phospholipids: C1 and C16 in TMA-DPH; C13 and C21 in Laurdan; N2 and C25 in PY3174; S1, N1, and C18 in di-8-ANEPPS.

To characterize the motional freedom of individual atoms in TMA-DPH, the mean squared displacement (MSD) of heavy atoms was calculated by exporting their coordinates from VMD^61^ and calculating the MSD according to eq 4 in MATLAB:

4 $$MSD\left(i\right)=\frac{\overset{N-i}{\underset{k=1}{\sum }}{∥c_{k+i}-c_{k}∥}^{2}}{N-i}$$

In eq 4, *c_j_* is a vector containing the *X*, *Y*, and *Z* coordinates of an atom in frame *j*, and *N* is the number of frames in the trajectory.

To calculate water penetration into the membrane, the positions of water and the phosphorus atom of phospholipids were exported using VMD. The position of the membrane–water interface was determined by fitting two parallel planes on the positions of phosphorus followed by calculating the distance of the oxygen of every intramembrane water molecule from the membrane–water interface followed by averaging these distances for every frame.

### Statistical Analysis

Measured data are represented as mean ± SEM obtained from *n* independent samples for spectrofluorometry or *n* individual cells from five independent experiments for confocal microscopy, as indicated in the figure legends. In spectrofluorometry, each sample contained approximately 100,000 cells. The *p*-values were calculated by Tukey’s HSD test carried out after significant differences were obtained for between-group effects in ANOVA. Differences were considered significant when *p* < 0.05 (*p < 0.05, **p < 0.01, ***p < 0.001, ****p < 0.0001).

## Results

### The Effects of Sterol-Cyclodextrin Complexes on the Fluorescence Anisotropy of TMA-DPH

In order to examine the effects of three different sterols, cholesterol (CHOL), 7-dehydrocholesterol (7DHC), and 6-ketocholestanol (6KC) on membrane biophysical parameters, we exogenously loaded CHO cells with these lipids for 1 h using their complexes with MβCD at a sterol concentration of 200 μM.^20,45^ As a control, cells were treated with native MβCD at a concentration equivalent to that of MβCD in the sterol-MβCD complexes. First, we examined the effects of these treatments on the fluorescence anisotropy of TMA-DPH that was quantified with spectrofluorometry as in our previous studies.^8–11,20^ Consistent with our expectations, all three examined sterols significantly increased TMA-DPH fluorescence anisotropy indicating reduced membrane fluidity when compared to the control, native MβCD-treated cells (Figure 1). The increases in anisotropy values were much larger in response to CHOL and 7DHC, which did not significantly differ from each other, whereas the 6KC-induced elevation was rather modest, significantly smaller, less than 20% of that induced by the other two sterols. Altogether, the TMA-DPH fluorescence anisotropy values for the different treatments were in the following order: CHOL ≅ 7DHC ≫ 6KC > MβCD.

**Figure 1:**
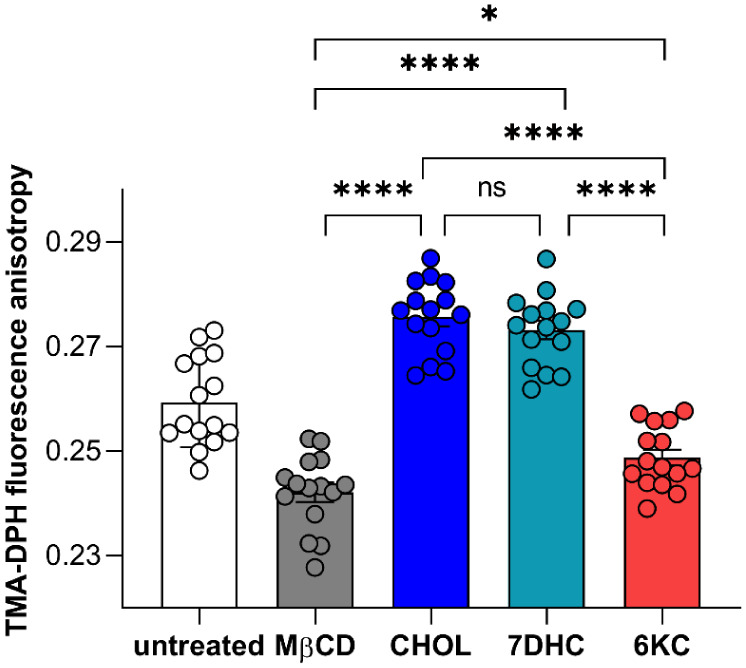
Effects of cyclodextrin-complexed sterols on the fluorescence anisotropy of TMA-DPH. Trypsinized CHO cells were incubated in normal Ringer’s solution or treated for 1 h with native MβCD, cholesterol-MβCD (CHOL), 7-dehydrocholesterol-MβCD (7DHC), or 6-ketocholestanol-MβCD (6KC) complexes. Cells were subsequently labeled with TMA-DPH, and the fluorescence anisotropy of the fluorophore negatively correlating with membrane fluidity was determined using spectrofluorometry. Individual TMA-DPH anisotropy values obtained in *n* = 15 independent samples containing approximately 100,000 cells, and their average values (±SEM) are plotted in the figure. Asterisks indicate significant differences between the samples (*p < 0.05 and ****p < 0.0001, ANOVA followed by Tukey’s HSD test).

### The Effects of Sterol-Cyclodextrin Complexes on the Generalized Polarization of Laurdan

Next, we investigated the alterations induced by 200 μM MβCD-complexed CHOL, 7DHC, and 6KC on the generalized polarization of Laurdan using conventional confocal microscopy with spectral imaging as in recent studies.^47–50^ The fluorophore was excited at 405 nm, and emitted intensities were detected in 8.9 nm intervals between 410 and 563 nm using a Quasar 32-channel GaAsP detector array (Figure S1A). A quantitative image analysis workflow (Figure 2A) was applied to determine the generalized polarization of Laurdan exclusively in the plasma membranes of individual cells. In accordance with results obtained with TMA-DPH, all three examined sterols resulted in significantly elevated Laurdan generalized polarization values negatively correlating with the extent of membrane hydration in comparison with control, MβCD-treated cells (Figure 2B). However, the relative efficiencies of sterols were slightly different when compared to alterations in TMA-DPH anisotropy. Although the CHOL-induced change in Laurdan generalized polarization was again the largest, the elevation induced by 7DHC was slightly but significantly smaller, ≈90% of that in response to CHOL. The 6KC-induced increase in the generalized polarization was likewise rather modest; however, it was relatively larger (≈40% of that induced by CHOL) than its effect on fluidity observed with TMA-DPH. In summary, the Laurdan generalized polarization values for the different treatments were in the following order: CHOL > 7DHC ≫ 6KC ≫ MβCD. Since 405 nm is generally not considered the optimal excitation for Laurdan^62^ and spectrofluorometry is the most widely applied technique to examine its generalized polarization, we also assessed the effects of sterols on this parameter using spectrofluorometry as in our previous studies.^8,10,11^ Notably, results obtained with this method exhibited strong quantitative consistency with those found with confocal microscopy (Figure S1B).

**Figure 2:**
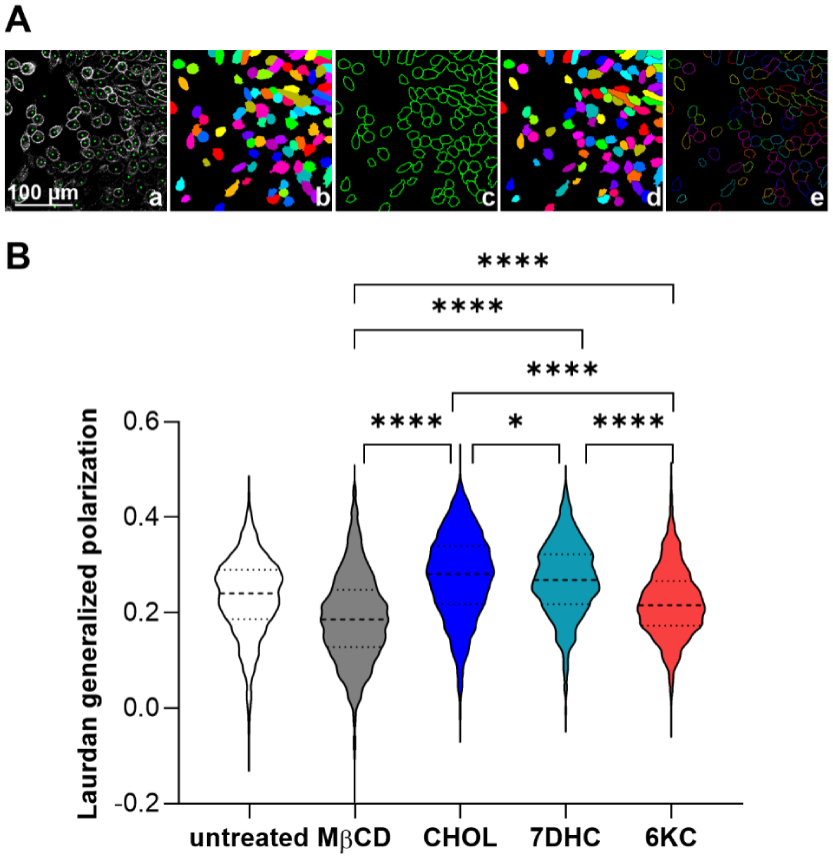
Effects of cyclodextrin-complexed sterols on the generalized polarization of Laurdan. (A) CHO cells grown onto an 8-well chambered coverglass were incubated in normal Ringer’s solution or treated for 1 h with native MβCD, cholesterol-MβCD (CHOL), 7-dehydrocholesterol-MβCD (7DHC), or 6-ketocholestanol-MβCD (6KC) complexes, and subsequently labeled with Laurdan. Confocal microscopic images were taken at the midplane of cells, and during quantitative image analysis, a custom-written manually seeded watershed algorithm (a) identified the cells (b) and the pixels corresponding to the plasma membrane of the cells (c). This was followed by the identification of individual cells (d), and, consequently, plasma membrane pixels of individual cells (e), and the generalized polarization of the fluorophore negatively correlating with the extent of membrane hydration was calculated on a pixel-by-pixel basis. (B) Violin plots were generated from median Laurdan generalized polarization values calculated exclusively from pixels corresponding to the plasma membrane for *n* = 2000–2500 individual cells per treatment condition obtained from five independent experiments, which also display median values with quartiles. Asterisks indicate significant differences between the samples (****p < 0.0001, ANOVA followed by Tukey’s HSD test).

### The Effects of Sterol-Cyclodextrin Complexes on the Generalized Polarization of PY3174

In the next step of our experiments, we explored changes exerted by CHOL, 7DHC, and 6KC on the generalized polarization of PY3174, which was originally proposed as a Laurdan substitute.^19^ Since due to its favorable spectral properties the dye is more suitable for conventional imaging techniques, we applied confocal microscopy to quantify the generalized polarization of the fluorophore as in our previous studies^11,20^ with slight modifications. The fluorophore was excited at 488 nm, and emitted intensities were detected in 8.9 nm intervals between 500 and 698 nm using a Quasar 32-channel GaAsP detector array (Figure S2A). The same quantitative image analysis workflow was applied to determine the generalized polarization of PY3174 exclusively in the plasma membranes of individual cells as in the case of Laurdan. While, just like in the case of Laurdan, all examined sterols significantly increased PY3174 generalized polarization, the relative efficiencies of sterols were substantially different (Figure 3). When compared to CHOL-induced effects, the elevation in response to 7DHC was significantly smaller, while the 6KC-induced increase was significantly greater, being ≈45% and ≈330% of that of CHOL, respectively. Thus, the PY3174 generalized polarization values for the different treatments were in the following order: 6KC ≫ CHOL > 7DHC > MβCD. Although the absolute PY3174 generalized polarization values slightly differed due to the use of a distinct detector system, quantitatively strongly consistent sterol-induced changes were found with spectrofluorometry as well (Figure S2B).

**Figure 3:**
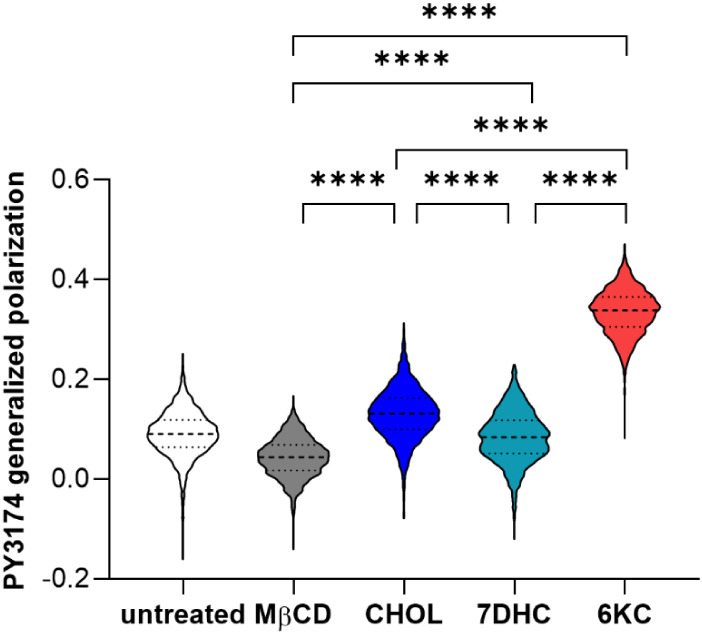
Effects of cyclodextrin-complexed sterols on the generalized polarization of PY3174. CHO cells grown onto an 8-well chambered coverglass were incubated in normal Ringer’s solution or treated for 1 h with native MβCD, cholesterol-MβCD (CHOL), 7-dehydrocholesterol-MβCD (7DHC), or 6-ketocholestanol-MβCD (6KC) complexes, and subsequently stained with PY3174. Confocal microscopic images were taken at the midplane of cells, and the generalized polarization of PY3174 was calculated on a pixel-by-pixel basis. Violin plots were generated from median PY3174 generalized polarization values calculated exclusively from pixels corresponding to the plasma membrane for *n* = 2500–3000 individual cells per treatment condition obtained from five independent experiments, which also display median values with quartiles. Asterisks indicate significant differences between the samples (****p < 0.0001, ANOVA followed by Tukey’s HSD test).

### The Effects of Sterol-Cyclodextrin Complexes on the Excitation Ratio of Di-8-ANEPPS

To explore alterations induced by CHOL, 7DHC, and 6KC on the membrane–water interface, we next tested their effects on the excitation ratio of di-8-ANEPPS positively correlating with the magnitude of dipole potential.^8,10,20,25,26^ For this, we applied confocal microscopy as in our previous studies.^10,25,26^ The dye was excited at 458 and 514 nm (Figure S3A), and the data analysis method introduced in Figure 2A was applied with the modification that here an excitation ratio was calculated instead of the generalized polarization. In these measurements, only 6KC and CHOL elevated the di-8-ANEPPS excitation ratio in the plasma membrane of cells and no significant changes were observed in response to 7DHC (Figure 4). The alteration induced by 6KC was significantly superior, ≈390%, in comparison with CHOL. In contrast, the effect of 7DHC was significantly lower, ≈8% of that after CHOL. In general, sterol-induced effects on the di-8-ANEPPS excitation ratio were similar to those observed in PY3174 generalized polarization measurements, with values for the different treatments being in the following order: 6KC ≫ CHOL > 7DHC ≅ MβCD. Measurements performed using spectrofluorometry provided quantitatively similar results regarding the effect of the different sterol derivatives (Figure S3B and C). Differences in the absolute values of the di-8-ANEPPS excitation ratio in the two experimental systems are due to the different ratios of the excitation intensities at 458 and 514 nm in microscopy and spectrofluorometry.

**Figure 4:**
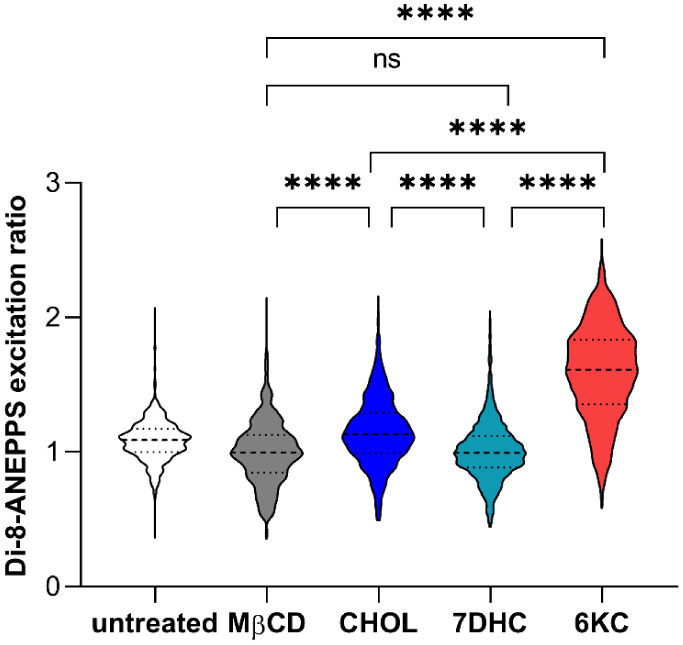
Effects of cyclodextrin-complexed sterols on the excitation ratio of di-8-ANEPPS. CHO cells grown onto an 8-well chambered coverglass were incubated in normal Ringer’s solution or treated for 1 h with native MβCD, cholesterol-MβCD (CHOL), 7-dehydrocholesterol-MβCD (7DHC), or 6-ketocholestanol-MβCD (6KC) complexes, and subsequently stained with di-8-ANEPPS. Confocal microscopic images were taken at the midplane of cells after excitation at two different wavelengths, and during quantitative image analysis, the median di-8-ANEPPS excitation ratio values of individual cells positively correlating with the magnitude of dipole potential were determined exclusively from data of pixels corresponding to the plasma membrane. Violin plots were generated from median di-8-ANEPPS excitation ratio values calculated exclusively from pixels corresponding to the plasma membrane for *n* = 1300–1700 individual cells per treatment condition obtained from five independent experiments, which also display median values with quartiles. Asterisks indicate significant differences between the samples (****p < 0.0001, ANOVA followed by Tukey’s HSD test).

### The Depth Localization of Membrane-Incorporated Environment-Sensitive Fluorophores

Our experimental results described in previous sections demonstrate that while the different sterols generally alter the fluorescence parameters of the four examined environment-sensitive fluorophores, there are notable and distinctive differences between the magnitudes of sterol-induced effects and the parameters of the dyes are not altered in parallel with each other. To investigate whether the distinct membrane localization of the fluorophores contributes to the observed differences, we performed comparative MD simulations with TMA-DPH, Laurdan, PY3174, and di-8-ANEPPS embedded into a POPC lipid bilayer (Figure 5). We generated a total of 12 solvated fluorophore–bilayer complex systems with the four fluorophores initially located at three different depths of the bilayer (Figure 6). After energy minimization and equilibration, production MD simulations were performed and atomic coordinates of all atoms were recorded every 10 ps. First, we examined the membrane thickness between the P atoms in the upper and lower layers for each system to demonstrate the overall stability. In all examined models, the thickness of the bilayer increased from the original 32 Å to approximately 38 Å within 30–50 ns, which was maintained in the remaining time of the simulations, suggesting the overall stability of the systems (Figure S4). While an analysis of time-dependent changes in tilt angles to the membrane normal (Figure S5) and positional RMSDs of heavy atoms (Figure S6) revealed considerable variations and, in one simulation, Laurdan underwent a flip motion similarly to that observed in a previous study,^63^ in general, all four fluorophores maintained stability in the bilayer and some general trends emerged in their membrane localization when compared to each other.

**Figure 5:**
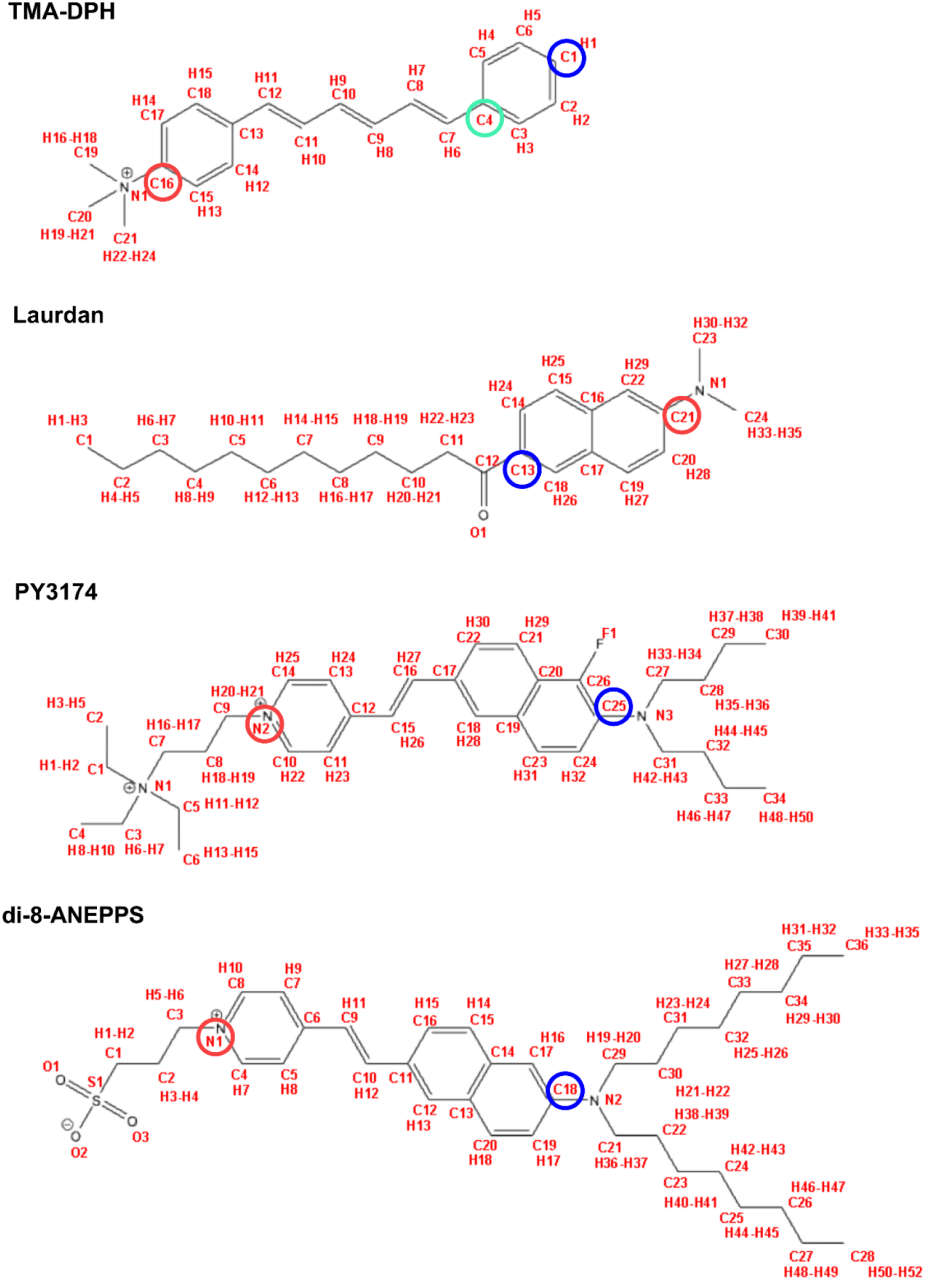
Schematic structures of the examined environment-sensitive fluorophores. Schematic structures of TMA-DPH, Laurdan, PY3174, and di-8-ANEPPS with the names of each atom labeled in red are displayed. The atoms selected for further analysis are circled in each fluorophore. The red and blue circles show the boundaries of the chromophore groups, whereas in TMA-DPH, the cyan and the blue circles together mark the extent of the nonammonium-bearing phenyl ring, which displays significantly higher mobility than the ammonium-bearing ring.

**Figure 6:**
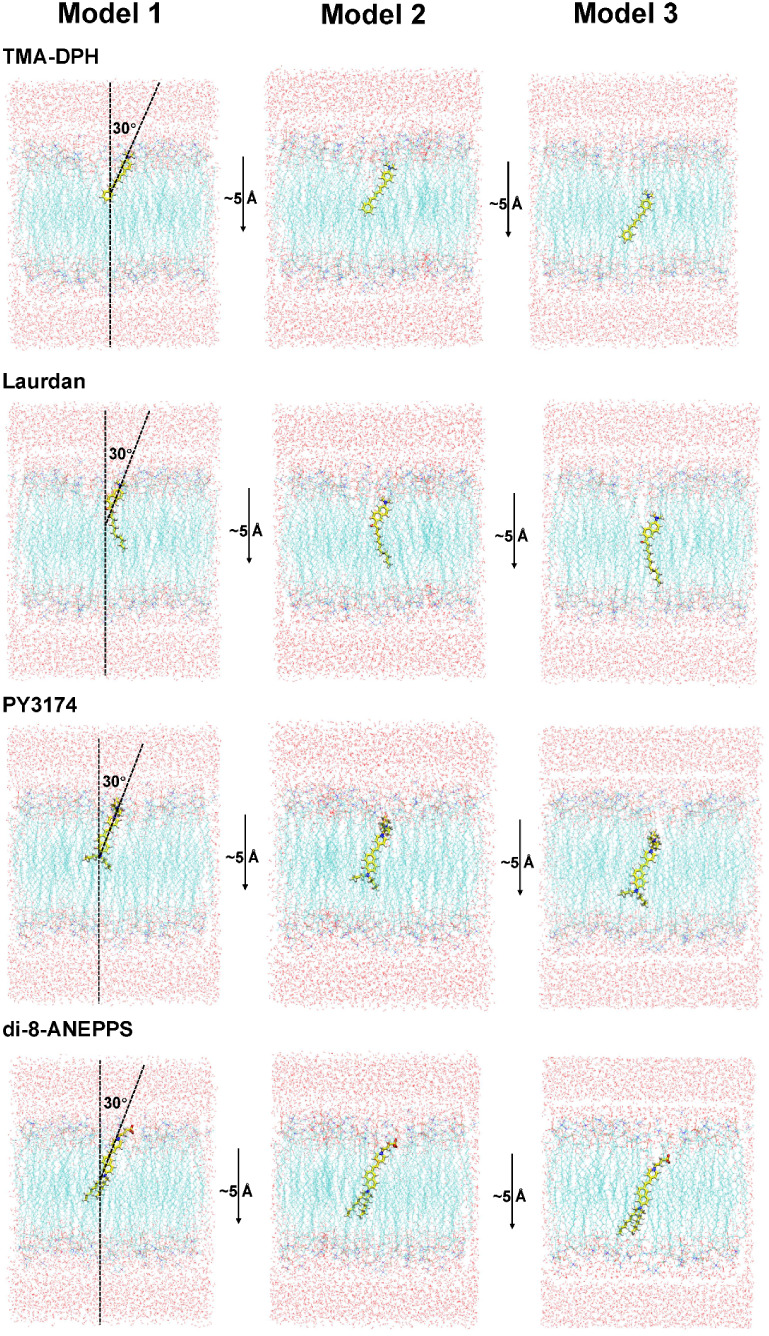
Representations of initial geometries of the examined environment-sensitive fluorophores. TMA-DPH, Laurdan, PY3174, and di-8-ANEPPS were embedded into a pre-equilibrated lipid bilayer composed of 1-palmitoyl-2-oleoyl-*sn*-glycero-3-phosphocholine (POPC) with the initial orientation of fluorophores having a 30° angle to the membrane normal. The tilt angle to membrane normal was measured based on the following selected atoms in each fluorophore molecule: N1 and C4 in TMA-DPH; N1 and C12 in Laurdan; N2 and N3 in PY3174; and N1 and N2 in di-8-ANEPPS. The tilt angle was labeled in Model 1. Model 1 for all four fluorophore molecules has the selected atoms (N1 in TMA-DPH; N1 in Laurdan; N2 in PY3174; N1 in di-8-ANEPPS) at the same depth from the phospholipid membrane surface, and in Models 2 and 3 of each fluorophore, the dye molecules were moved toward the center of the bilayer by 5 Å and 10 Å, respectively.

To investigate the depth localization of the environment-sensitive dyes, we followed time-dependent changes in the distance of their selected atoms from the P atoms of phospholipids at the proximal interface. During the analysis, first we examined atoms at the two opposing edges of the chromophore moiety of the fluorophores: C1 and C16 in TMA-DPH; C13 and C21 in Laurdan; N2 and C25 in PY3174; N1 and C18 in di-8-ANEPPS (Figure 5). Distinctive depths of incorporation are obvious in both the representative snapshots of the simulation (Figure 7A) and the time-dependence of the distance of selected atoms from the membrane–water interface (Figure 7B). Furthermore, to characterize the depth localization quantitatively, we calculated the average position of the selected atoms in the last 100 ns intervals of each simulation with the fluorophores (Figure 7C). In accordance with experimental results and our hypothesis, the chromophore groups of TMA-DPH and Laurdan were found to be the deepest in the bilayer. However, somewhat surprisingly at first glance, the fluorophore moiety of TMA-DPH was a bit shallower than that of Laurdan with average distances of 9.0 and 10.6 Å from the interface, respectively. In contrast, the chromophore of PY3174 was localized a lot more superficially with an average position of 4.7 Å from the interface. The long chromophore group of di-8-ANEPPS resides, on average, 7.3 Å from the interface, that is, somewhere between the average depths of Laurdan and PY3174. Altogether, the localization of the fluorescent moieties only partially explained our experimental results pointing at the need for considering additional molecular details in terms of the sensitivity of the dyes to the local microenvironment.

**Figure 7:**
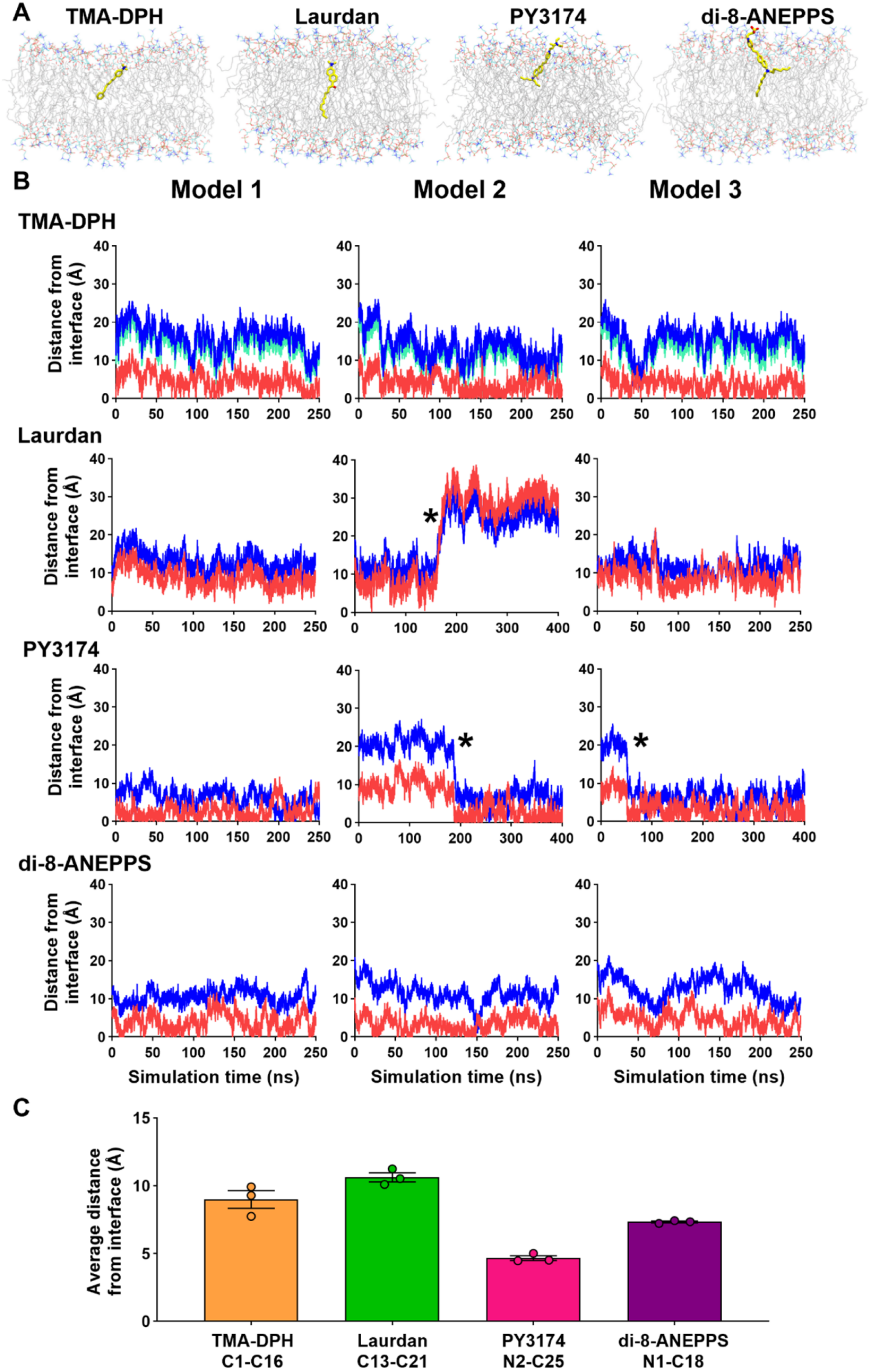
Membrane localization of the chromophore moieties of the examined environment-sensitive fluorophores. TMA-DPH, Laurdan, PY3174, and di-8-ANEPPS were embedded into a pre-equilibrated lipid bilayer composed of 1-palmitoyl-2-oleoyl-*sn*-glycero-3-phosphocholine (POPC) at three different depths (Model 1, Model 2, and Model 3), and after energy minimization and equilibration, production MD simulations were run for 250–400 ns. (A) Representative snapshots from the simulations demonstrate the typical localization of the fluorophores. (B) Distances of the selected atoms in each fluorophore (C1, C4, and C16 in TMA-DPH; C13 and C21 in Laurdan; N2 and C25 in PY3174; N1 and C18 in di-8-ANEPPS) were calculated to the membrane surface defined as the position of the P atoms in phospholipids and plotted as a function of simulation time. For the analysis, atoms were selected at the two edges of the chromophore moiety facing and opposing the membrane–water interface, and their distances are plotted in red and blue, respectively. In the case of TMA-DPH, the time-dependent distance between the interface and the C4 on the opposing edge of the mobile group of TMA-DPH mainly responsible for its rotation is plotted in cyan. The asterisks represent the flip motion of Laurdan into the opposing bilayer leaflet and large-scale positional changes of PY3174. (C) To characterize the depth localization quantitatively, we calculated the mean position of the selected atom pairs and determined the average of these values in the last 100 ns intervals of simulations. The calculated average distance values from the interface in *n* = 3 independent simulations, and their mean (±SEM), are plotted in the panel.

The sensitivity of a fluorophore to membrane biophysical parameters is not necessarily exclusively determined by the localization of its chromophore moiety. For example, in the case of TMA-DPH, its fluorescence anisotropy, that is, its rotational freedom, is examined. During the rotational motion of the probe, its charged trimethylammonium group is localized near the more ordered membrane–water interface at a relatively fixed position with restrained mobility (Figure 8A) due to strong interactions with the neighboring chemical groups of phospholipids and can thus be considered a pivot around which the rotation occurs, a conclusion supported by its substantially lower mean-squared displacement compared to the other, phenyl group. The elongated rod-like part of the molecule contains conjugated double bonds making the structure rigid, and the molecule ends in a ring complex that exhibits the largest motional freedom (Figure 8A). The latter part of the dye seems to be the main determinant of its rotation for two reasons. First, it is the most distant moiety of the molecule from the pivot and, therefore, can generate the largest torque arising from the collision of an atom of a lipid molecule with TMA-DPH. Second, this portion is localized in the least ordered inner layers of the membrane, favoring the rotation. Consequently, the fluorescence anisotropy of TMA-DPH may mostly depend on the structural organization of the membrane layer in which the nonammonium-bearing ring is found.

**Figure 8:**
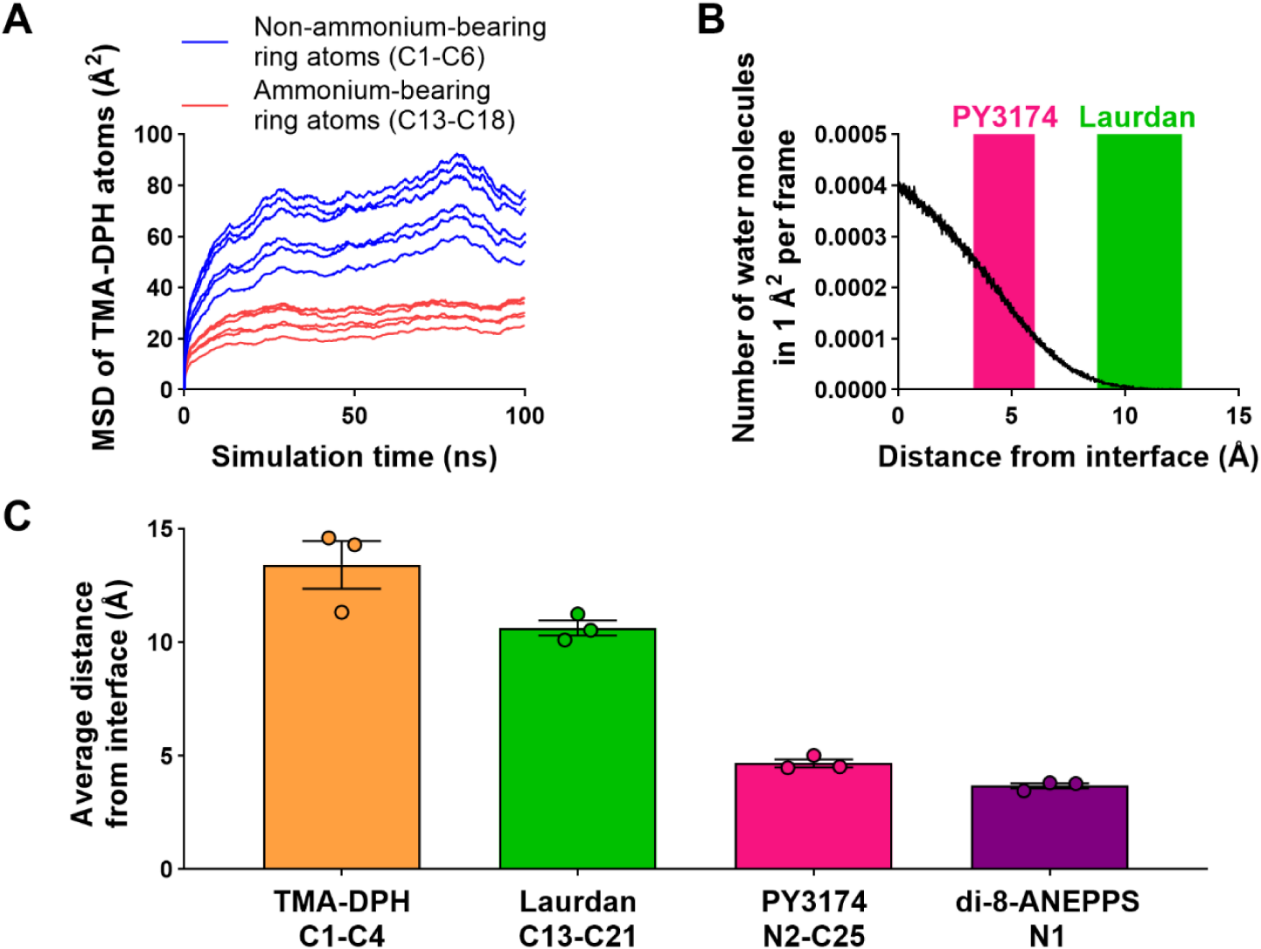
Membrane localization of the sensor regions of the examined environment-sensitive fluorophores. TMA-DPH, Laurdan, PY3174, and di-8-ANEPPS were embedded into a pre-equilibrated lipid bilayer composed of 1-palmitoyl-2-oleoyl-*sn*-glycero-3-phosphocholine (POPC) at three different depths (Model 1, Model 2, and Model 3), and after energy minimization and equilibration, production MD simulations were run for 250–500 ns. (A) The mean squared displacement of heavy atoms of the ammonium-bearing and the nonammonium-bearing ring of TMA-DPH was determined and plotted as a function of simulation time to quantitate the motional freedom of the different molecular moieties within the first 100 ns. (B) The average number of water molecules penetrating the layers of the membrane at different depths was determined and plotted as a function of distance from the membrane–water interface. The extent of water penetration did not show substantial time-dependent variations in the trajectories. Pink and green shaded areas represent the average positions of the hydration-sensitive chromophore moieties of PY3174 and Laurdan, respectively. (C) To characterize the depth localization of the environment-sensitive moieties of the fluorophores quantitatively, we calculated the mean position of the selected atoms corresponding to the sensor regions of the probes and determined the average of these values in the last 100 ns intervals of simulations. The environment-sensitive group of TMA-DPH is the nonammonium-bearing phenyl ring, the chromophores between C13–C21 and N2–C25 in Laurdan and PY3174, respectively, and the N1 atom in di-8-ANEPPS. The calculated average distance values from the interface in *n* = 3 independent simulations, and their mean (±SEM), are plotted in the panel.

Due to the solvent relaxation mechanism, the spectral properties of Laurdan and PY3174 change in response to the hydrophobicity of the local microenvironment and thus may rather sense the amount of water molecules penetrating the membrane layers surrounding the chromophore moiety itself (Figure 8B). In contrast, the voltage-dependent behavior of di-8-ANEPPS, similarly to other related fluorophores in the family and other push–pull-type dyes, originates from different dipole moments of the ground and photoexcited states through a large change in the electronic structure, which occurs due to excitation. In the resting state, the positive charge of the probe resides on the pyridinium N1 nitrogen at the hydrophilic end, which moves across the long axis of the probe to the anilino N2 nitrogen at the hydrophobic end of the molecule upon photoexcitation. The energy required for this charge shift depends on the local electric field resulting in a shift in the excitation spectrum of the probe when the dipole potential changes.^64,65^ It was also suggested that the excitation energy varies primarily according to the stabilization of the ground state, that is, when the charge is localized on N1,^65^ which is found at the steepest part of the dipole potential gradient, i.e., the region at which the largest electric field arises and is thus the main determinant of electric field sensitivity.^5,6,66^ Consequently, the environment sensor moiety is different for the probes: in TMA-DPH, the nonammonium-bearing ring bounded by C1 and C4; in Laurdan and PY3174, the chromophores between C13–C21 and N2–C25, respectively; while the N1 atom in di-8-ANEPPS. Therefore, we compared the average positions of these sensor regions of the probes in the last 100 ns intervals of simulations (Figure 8C). As expected based on our experimental results, the sensor of TMA-DPH was found in the hydrophobic core of the bilayer, deeper than that of Laurdan, which were on average 13.4 and 10.6 Å from the interface, respectively. When comparing the two superficial probes, PY3174 and di-8-ANEPPS, the environment-sensitive moiety of the latter was localized closer to the interface with an average position of 3.7 Å from the membrane–water boundary as opposed to a distance of 4.7 Å for PY3174. In summary, MD simulations in a simple model bilayer supported our hypothesis that the dyes exhibit distinctive sensitivity to the different aspects of the structural organization of biological membranes due to their characteristic depth localization.

## Discussion

As the major finding of our study, using various sterol-MβCD complexes and fluorescence-based measurement techniques, we reveal that the commonly applied environment-sensitive dyes, including TMA-DPH, Laurdan, PY3174, and di-8-ANEPPS, report on distinct aspects of the molecular organization of cellular membranes. Our comparative experimental results imply that the three most widely examined membrane biophysical parameters, fluidity, hydration, and dipole potential, are not equivalent and alterations in the lipid composition of biological bilayers, as opposed to what is assumed often in the literature, can in fact change in an incongruent manner. Furthermore, by comparative MD simulations in a simple model bilayer, we also demonstrate that the four examined fluorophores may be sensitive to different aspects of membrane organization due to their distinctive incorporation into membranes at various depths. Based on our results, we propose that lipid-induced alterations in membrane structure can be appropriately described in living cells only by a combination of various environment-sensitive fluorophores, which would indeed help in understanding the molecular details and (patho)­physiological roles of membranes from a biophysical point of view.

Biological membranes comprise a compositionally and biophysically complex lipid matrix to solvate membrane proteins and exert substantial modulatory actions on their structure and function both via direct lipid–protein interactions and indirectly through modifying bulk, molecular order-associated membrane biophysical properties.^1,2^ In living cells, the latter are characterized by fluidity, hydration, and dipole potential that are defined as the motional freedom of membrane constituents, the extent of water penetration into the membrane, and a large positive intramembrane potential generated by the nonrandom orientation of molecular dipoles of membrane constituents, respectively. While these parameters are definitely linked to each other, based on theoretical considerations and experimental data, we proposed recently that they describe the membrane structure at different depths. Namely, fluidity may characterize mainly the hydrophobic center of bilayers, hydration reports on the layers between the core and interfacial regions, whereas dipole potential characterizes the membrane–water interface.^2–4,7^ While this distinctive classification is didactically expedient, it has not been experimentally and comparatively justified previously. In this paper, we set out to prove this hypothesis by using environment-sensitive fluorophores that previously *de facto* formed the basis for the distinctive operational definition of these properties. Membrane fluidity was quantified by following the fluorescence anisotropy of TMA-DPH using spectrofluorometry, which characterizes the rotational freedom of the dye by determining its reorientation during the excited-state lifetime as a function of local microviscosity.^8–13,20^ Membrane hydration was investigated by confocal microscopy and spectrofluorometry with Laurdan as its emission spectrum is red-shifted in a hydrophilic environment due to solvent relaxation, which is quantified by its generalized polarization when measuring emission at two different wavelengths after excitation at a single wavelength.^8,9,11,14–18,47,48,50^ Alternatively, hydration was measured with a microscopy-based approach using PY3174 that was initially proposed as a promising Laurdan-substituting candidate with more favorable spectral characteristics more suitable for conventional confocal microscopy.^11,19,20^ The magnitude of dipole potential was investigated using confocal microscopy and di-8-ANEPPS that exhibits electrochromism, i.e., changes in spectral properties as a function of the strength of the local intramembrane electric field, and thus can be utilized in an excitation ratiometric assay quantifying the ratio of photons emitted in the same wavelength range after excitation at two different wavelengths.^8,10,20–29^

Given that the biophysical properties of bilayers essentially depend on the quality and quantity of membrane lipids, and in particular that of sterols, we manipulated cell membrane sterol levels by applying MβCD alone or precomplexed with CHOL, 7DHC, or 6KC to elicit lipid-induced changes in the membrane biophysical properties and thus the fluorescence parameters of TMA-DPH, Laurdan, PY3174, and di-8-ANEPPS. From among various βCDs utilized in a wide variety of experimental, pharmaceutical, and clinical applications,^40,42^ we chose MβCD due to its highest *in vitro* efficiency to form complexes with sterols.^43,44^ The native MβCD is capable of depleting cell membrane cholesterol^43,44^ whereas, when precomplexed, it can effectively load the cell membrane with various sterols including CHOL, 7DHC, and 6KC as well.^20,28,44–46^ However, in these applications, it should be kept in mind that precomplexation and equilibrating experimental conditions result in a mixture of native, empty MβCD and MβCD-sterol complexes and, therefore, two processes simultaneously occur during incubation, i.e., the empty MβCD depletes cholesterol, while the precomplexed MβCD increases membrane sterol abundance. As a result, the applied concentration of the CD, the relative concentration of sterols in the complex, and the initial membrane cholesterol level together determine the resulting effect of the treatment. Therefore, treatment with empty MβCD at a CD concentration equivalent to that in the MβCD-sterol complexes should be applied as a control.^20,28,44,46^ Using this experimental approach, we found that, in general, all of the three examined sterols resulted in significant changes in all examined fluorescence parameters. However, large differences were observed in the relative efficiencies of sterols in the different parameters. While all sterols increased TMA-DPH fluorescence anisotropy values indicating reduced fluidity, CHOL- and 7DHC-induced changes did not significantly differ from each other and were significantly larger than those after 6KC, which were rather moderate (Figure 1). All sterols resulted in significant elevations in Laurdan generalized polarization mirroring an elevated hydrophobicity. However, here the strengths of the effects decreased in the order CHOL > 7DHC > 6KC, and each pair differed significantly (Figure 2). When examining the supposedly Laurdan-substituting PY3174, striking differences were seen. Although all sterols increased PY3174 generalized polarization values, by far the largest change was seen after 6KC, the effect of CHOL was significantly smaller, and that of 7DHC was the smallest, albeit still statistically significant (Figure 3). In measurements with di-8-ANEPPS, 6KC resulted in the highest excitation ratios, CHOL induced smaller but still significant increases, while 7DHC failed to result in any statistically significant effect (Figure 4). The results obtained with PY3174 showed much better correlation with those of di-8-ANEPPS and were remarkably different from those seen with Laurdan. This might be surprising considering the relevant literature. PY3174 was originally described to report on “lateral packing” and “lipid order” and, while the exact sensing mechanism was not discussed, it was proposed as a functional Laurdan analogue.^19^ Subsequently, although sporadic studies mentioned “voltage-sensitive” PY3174,^67,68^ which would imply similarity with the structural analogue di-8-ANEPPS, recent impactful publications termed “polarity-sensitive” PY3174 and applied it as a Laurdan alternative without mentioning plausible voltage sensitivity, dipole potential sensitivity, or any potentiometric mechanism.^69,70^

Limitations of our experimental approach include that data obtained with the most commonly utilized methods, that is spectrofluorometry and confocal microscopy, were compared. Hence, one might argue that merely using distinct measurement techniques would be responsible for the incongruence of probe responses through membrane changes induced by trypsinization, lack of adherence, or dye internalization. To rule out the major contribution of trypsinization- and adherence-related effects, we repeated the measurements with Laurdan, PY3174, and di-8-ANEPPS using spectrofluorometry and the both qualitatively and quantitatively congruent results obtained strongly supported the conclusions based on confocal microscopy (Figures S1–S3). Internalization might be a relevant problem for the interpretation of our results since internalized dye molecules localized in intracellular membranes containing far lower sterol levels would experience much smaller environmental differences than in the plasma membrane in response to our treatments. However, representative confocal microscopic images argue against large-scale internalization of the probes at the time scale of our measurements (Figures S1–S3). Moreover, it is reasonable to assume that qualitative differences, that is, the efficiency order of the different sterols, should not be sensitive to internalization. In accordance, while numeric changes in dye parameters cannot be translated into quantitative membrane biophysical properties, for instance, variation in di-8-ANEPPS excitation ratios into mV changes of dipole potential, “semiquantitative” data with prominent changes in the efficiency order of sterols convincingly support the distinct behavior of the dyes.

The observation that sterols exhibited distinctive relative efficiencies to modify TMA-DPH fluorescence anisotropy, Laurdan generalized polarization, PY3174 generalized polarization, and di-8-ANEPPS excitation ratio values unequivocally points at different membrane biophysical properties monitored by these fluorescence parameters. Although this is not fully unexpected in light of sporadic studies carried out with a combination of these fluorophores demonstrating dissimilar lipid alteration-induced changes in the examined parameters,^8,10,20^ just as observed in fluorescence lifetime changes of other environment-sensitive probes,^71–73^ a rigorous comparative analysis elucidating their interrelationship has not been performed yet, and it is still generally assumed that they change in parallel with each other. Actually, several previous reports have already hinted at the distinctive localization of these dyes in the bilayers. MD simulations showed that the fluorophore group of TMA-DPH, in spite of carrying a hydrophilic trimethylammonium group, is buried deep inside the hydrophobic core.^74^ In the case of Laurdan, fluorescence spectra analysis in solid-supported lipid bilayers showed that Laurdan is incorporated into membranes in a way that its fluorescent moiety is localized deeper below the glycerol backbone of the phospholipids near the sn-1 carbonyls,^75^ and a similar localization was proposed by MD simulations as well.^63,76^ As for di-8-ANEPPS, measurements with the parallax method in model bilayers showed a localization of its fluorescent moiety exactly at the membrane–water interface,^24^ and MD simulations also demonstrated the localization of the polar group of the dye in the headgroup region of phospholipids.^66,77,78^ On the other hand, the molecular details of membrane incorporation of PY3174 have not been examined before. Furthermore, all the above studies were carried out with only one of the four environment-sensitive dyes, and accordingly, a comprehensive and comparative MD simulation analysis is completely missing.

In accordance, to shed light on molecular details that may explain their different behavior, we performed comparative MD simulations with TMA-DPH, Laurdan, PY3174, and di-8-ANEPPS embedded in a simple POPC bilayer. We analyzed the membrane incorporation of these fluorophores with a special emphasis on the exact localization of their environment-sensitive moieties. Consistent with our experimental results and previous simulations,^66,74,75,77,78^ we observed distinctive bilayer localization of the dyes. Namely, the chromophore moieties of TMA-DPH and Laurdan are found in the deep hydrophobic core regions of the bilayer, on average 9.0 Å and 10.6 Å from the interface, respectively, whereas those of PY3174 and di-8-ANEPPS are a lot more peripheral with mean positions of 4.7 Å and 7.3 Å, respectively, when compared to the membrane–water boundary (Figure 7). This implies that, as opposed to its original description of being a presumable Laurdan alternative, the membrane localization of PY3174 is notably different with its chromophore region residing at a lot shallower position when compared to Laurdan. While the mean localization of the chromophore group of Laurdan and PY3174 determines their sensitivity to the local microenvironment, the sensing mechanisms of TMA-DPH and di-8-ANEPPS are extensively different. The rotational freedom and hence the fluorescence anisotropy of TMA-DPH mainly depend on the nonammonium-bearing phenyl ring of the probe, and therefore, it is predominantly determined by the location of this moiety, which was found on average 13.4 Å from the interface (Figure 8). In contrast, the N1 atom responsible for the voltage sensitivity of di-8-ANEPPS is superficial, very close to the interface with an average position of 3.7 Å from it. Hence, the dye primarily detects changes in the structural organization at the interfacial region. Altogether, our MD simulation results are consistent with our experimental findings and provide an explanation for the different sensitivity of these fluorophores to the different membrane biophysical properties due to the distinctive depth localization of the dyes (Figure 9). Furthermore, both our simulation and experimental data emphasize that PY3174, unlike its suggested analogue compound Laurdan, reports on the structural organization of more peripheral bilayer regions. Therefore, the information provided by PY3174 resembles much more that of di-8-ANEPPS rather than Laurdan.

**Figure 9:**
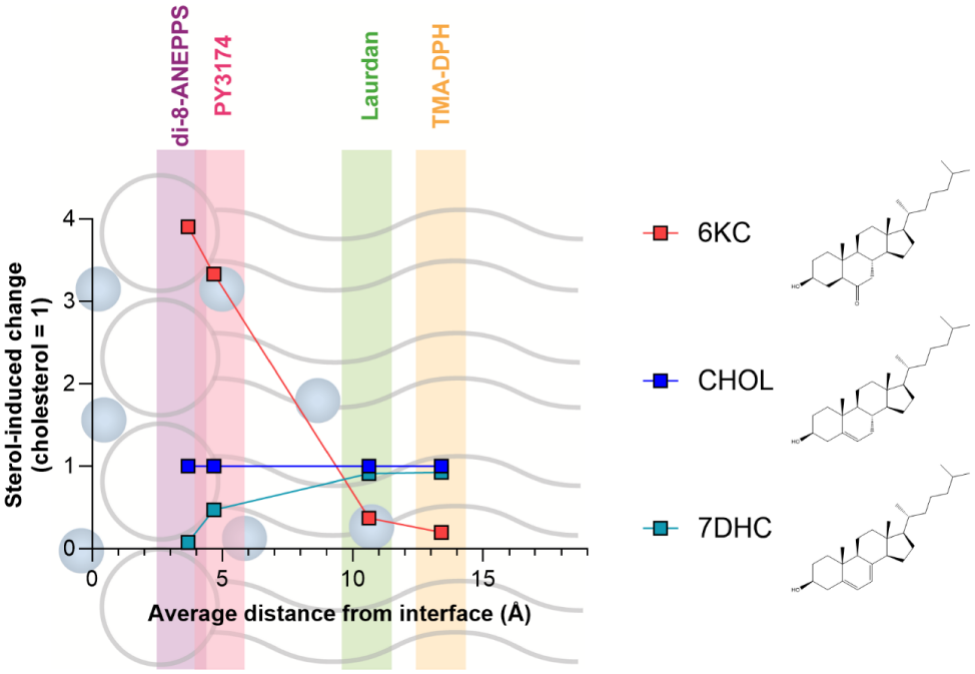
Effects of cyclodextrin-complexed sterols on fluorescent parameters of environment-sensitive fluorophores reporting on biophysical properties of bilayers at different depths. Effects of cholesterol-MβCD (CHOL), 7-dehydrocholesterol-MβCD (7DHC), or 6-ketocholestanol-MβCD (6KC) complexes on TMA-DPH fluorescence anisotropy, Laurdan generalized polarization, PY3174 generalized polarization, and di-8-ANEPPS excitation ratio were experimentally quantified in living cells as described previously, and subsequently normalized to changes induced by CHOL in the given parameter. The average distance values of the sensor moieties of the fluorophores from the membrane–water interface of 1-palmitoyl-2-oleoyl-*sn*-glycero-3-phosphocholine (POPC) bilayers were determined with MD simulations. Normalized changes in fluorescence parameters were then plotted as a function of the distance from the interface to demonstrate the relative efficiencies of the sterols to induce alterations in membrane biophysical properties at various depths.

While, to our knowledge, no such detailed analysis of the membrane biophysical effects of CHOL, 7DHC, and 6KC has been performed previously, our findings are in general agreement with partial results of literature data, mainly obtained in model membranes. It is well-documented that an increased membrane CHOL abundance reduces membrane fluidity by inducing stretching of phospholipid acyl chains and decreasing the average cross-sectional area per lipid,^11,12,20,30,33–35^ decreases hydration by lowering the penetration of water molecules,^11,14–17,20^ and elevates the magnitude of dipole potential due to its intrinsic dipole moment, condensation of the phospholipid headgroup region, and changing the amount and orientation of membrane-associated water molecules.^8,20,22–24,26,27,36,37^ In most aspects, 7DHC, having merely an extra double bond at the seventh position in the sterol ring, exerts generally similar biophysical actions on membrane fluidity and hydration.^20,39^ On the other hand, its dipole potential-increasing effects are much smaller than those of CHOL.^20,24,28^ This may result from differences in dipole moment, orientation (tilt angle), or the extent of association of water molecules with the membrane.^24^ Contrastingly, 6KC is the most widely used compound to efficiently elevate the magnitude of dipole potential,^8,20,21,23,25^ without inducing notable changes in fluidity or hydration,^8,20^ suggesting its exquisite actions on the membrane–water interface. Altogether, literature data and, particularly, our comparative experimental results together with MD simulations of the applied environment-sensitive fluorophores unequivocally demonstrate that while CHOL affects molecular organization throughout the whole width of biological membranes, 7DHC and 6KC predominantly alter the hydrophobic core and the interfacial regions, respectively (Figure 9). The distinctive effects of these sterols can provide invaluable tools to elucidate the mechanisms of indirect lipid-mediated modulation of protein function when applied in combination in experiments for identifying the molecular region of proteins affected by membrane biophysics.

As mentioned above, changes in membrane biophysical properties can mediate the effect of alterations in membrane lipid composition on the function of membrane proteins.^1,2^ For example, the packing order in the acyl chain regions of phospholipids can modulate protein function by modifying the state of aggregation, rate of conformational changes, secondary structure, and relative stability of certain protein conformers with different functional activities according to the hydrophobic mismatch and elastic coupling theories through altering hydrophobic thickness, elastic properties, and spontaneous curvature of bilayers as reviewed recently.^2,3,7,79,80^ Furthermore, the interaction of charged or polar residues of membrane proteins with the large electric field associated with the dipole potential at the membrane–water interface can alter the conformational stabilities thereby shifting their equilibrium.^2,5,6,81^ Accordingly, lipid-induced alterations in membrane biophysical properties were previously shown to affect the mechanosensation of various ion channels including Piezo and volume-regulated anion channels,^82,83^ the inactivating behavior of Piezo1,^84^ and the voltage sensitivity and activation gating of voltage-gated potassium channels.^10,45,85^ Indirect lipid actions were also described in ATP-driven pumps to influence ATPase and pumping activity of Na^+^-K^+^ ATPase^86^ and P-glycoprotein.^22^ Besides transporters, membrane biophysics affects the functioning of various cell surface receptors as well. For example, the local bilayer microenvironment contributes to the selection of a preferred conformation from various possible homo- and heteromeric associates of ErbB proteins^87,88^ and other receptor tyrosine kinases as well,^89^ which can manifest in altered ligand binding, association, autophosphorylation, and downstream signaling.^25,90,91^ In addition, lipids were shown to affect ligand binding and functional activity of various G protein-coupled receptors including nicotinic acetylcholine receptors,^92^ metabotropic glutamate receptors,^93^ β-adrenergic receptors,^94^ and serotonin receptors.^95,96^ Indirect lipid modulation of protein function is of outstanding biological relevance due to the intrinsic qualitative and quantitative membrane lipid alterations in a multitude of human pathological conditions such as metabolic diseases,^97–99^ neurodegeneration,^100,101^ lysosomal storage disorders,^102^ and tumors.^4,103–105^ Therefore, elucidating molecular mechanisms of membrane biophysics-related phenomena is of crucial importance both in regard to understanding disease pathomechanisms and the development of novel therapeutic approaches, and the combined application of environment-sensitive fluorophores and sterols eliciting distinctive membrane biophysical effects can be invaluable tools in the future for this purpose.

## Conclusion

In conclusion, we experimentally demonstrated in living cells that the fluorescence parameters of different environment-sensitive dyes—although generally assumed to provide equivalent information about “membrane order”—in fact change incongruently in response to various sterols. Our comparative MD simulations show that this is due to the distinct depth localization of the sensor moieties of the probes within bilayers. Our results suggest that membrane structure can be adequately described only with a combination of such fluorophores that characterize the molecular organization of bilayers at different depths.

## Supplementary Material



## Notes

Six figures demonstrating representative spectral images of Laurdan and sterol effects on its generalized polarization using spectrofluorometry (Figure S1), representative spectral images of PY3174 and sterol effects on its generalized polarization using spectrofluorometry (Figure S2), representative confocal images of di-8-ANEPPS and sterol effects on its excitation spectrum and excitation ratio using spectrofluorometry (Figure S3), time-dependent changes in the bilayer thickness (Figure S4), dye orientation (Figure S5), and positional variation of dye heavy atoms (Figure S6) during the MD simulations (PDF)

## Abbreviations

6KC: 6-ketocholestanol
7DHC: 7-dehydrocholesterol
CD: cyclodextrin
CHOL: cholesterol
di-8-ANEPPS: 4-(2-[6-(dioctylamino)-2-naphthalenyl]­ethenyl)-1-(3-sulfopropyl)­pyridinium inner salt
Laurdan: 6-dodecanoyl-*N*,*N*-dimethyl-2-naphthylamine
MβCD: methylated β-cyclodextrin
POPC: 1-palmitoyl-2-oleoyl-*sn*-glycero-3-phosphocholine
PY3174: (4-[2-(6-dibutylamino-5-fluoro-naphthalen-2-yl)-vinyl]-1-(3-triethylammonio-propyl)-pyridinium dibromide
TMA-DPH: 4′-(trimethylammonio)-diphenylhexatriene
