# Supplementary material for: The Many Layers of Membrane Biophysics: Environment-Sensitive Fluorophores Report on Structural Organization of Biological Membranes at Various Depths
Source: Anal Chem. 2026 Jun 8;98(24):17638–54. doi: 10.1021/acs.analchem.5c07236 (PMC13295090; doi:10.1021/acs.analchem.5c07236)
Supplement: Supplementary file 1 [file ac5c07236_si_001.pdf]

SUPPORTING INFORMATION

The many layers of membrane biophysics: Environment-sensitive fluorophores report on structural organization of biological membranes at various depths

*Florina Zakany[a], Rosemary Chandrakanthi Kothalawala[a], Olivér Pavela[b,c], Lajos Szente[d], Zoltan Varga[a], Gyorgy Panyi[a], Tamás Beke-Somfai[b], Peter Nagy[a], Tamas Kovacs[a]**

[a] Department of Biophysics and Cell Biology, Faculty of Medicine, University of Debrecen and MTA Centre of Excellence, Hungarian Academy of Sciences, Egyetem tér 1, Debrecen H-4032, Hungary

[b] Biomolecular Self-assembly Research Group, Institute of Materials and Environmental Chemistry, HUN-REN Research Centre for Natural Sciences, Magyar Tudósok Körútja 2, Budapest H-1117, Hungary

[c] Hevesy György PhD School of Chemistry, Eötvös Loránd University, Pázmány Péter Sétány 1/A, Budapest H-1117, Hungary

[d] CycloLab Cyclodextrin R&D Laboratory Ltd., Illatos u. 7., Budapest H-1097, Hungary

[*] corresponding author, kovacs.tamas@med.unideb.hu

Summary: 10 Pages and 6 Figures

Table of Contents

Figure S1. Examination of effects of cyclodextrin-complexed sterols on the generalized polarization of Laurdan using confocal microscopy and spectrofluorometry S3

Figure S2. Examination of effects of cyclodextrin-complexed sterols on the generalized polarization of PY3174 using confocal microscopy and spectrofluorometry S5

Figure S3. Examination of effects of cyclodextrin-complexed sterols on the excitation ratio of di-8-ANEPPS using confocal microscopy and spectrofluorometry S7

Figure S4. Time-dependent changes in the bilayer thickness during the MD simulations........... S8

Figure S5. Time-dependent changes of dye orientation during the MD simulations S9

Figure S6. Time-dependent changes in the positional variation of dye heavy atoms during the MD simulations S10

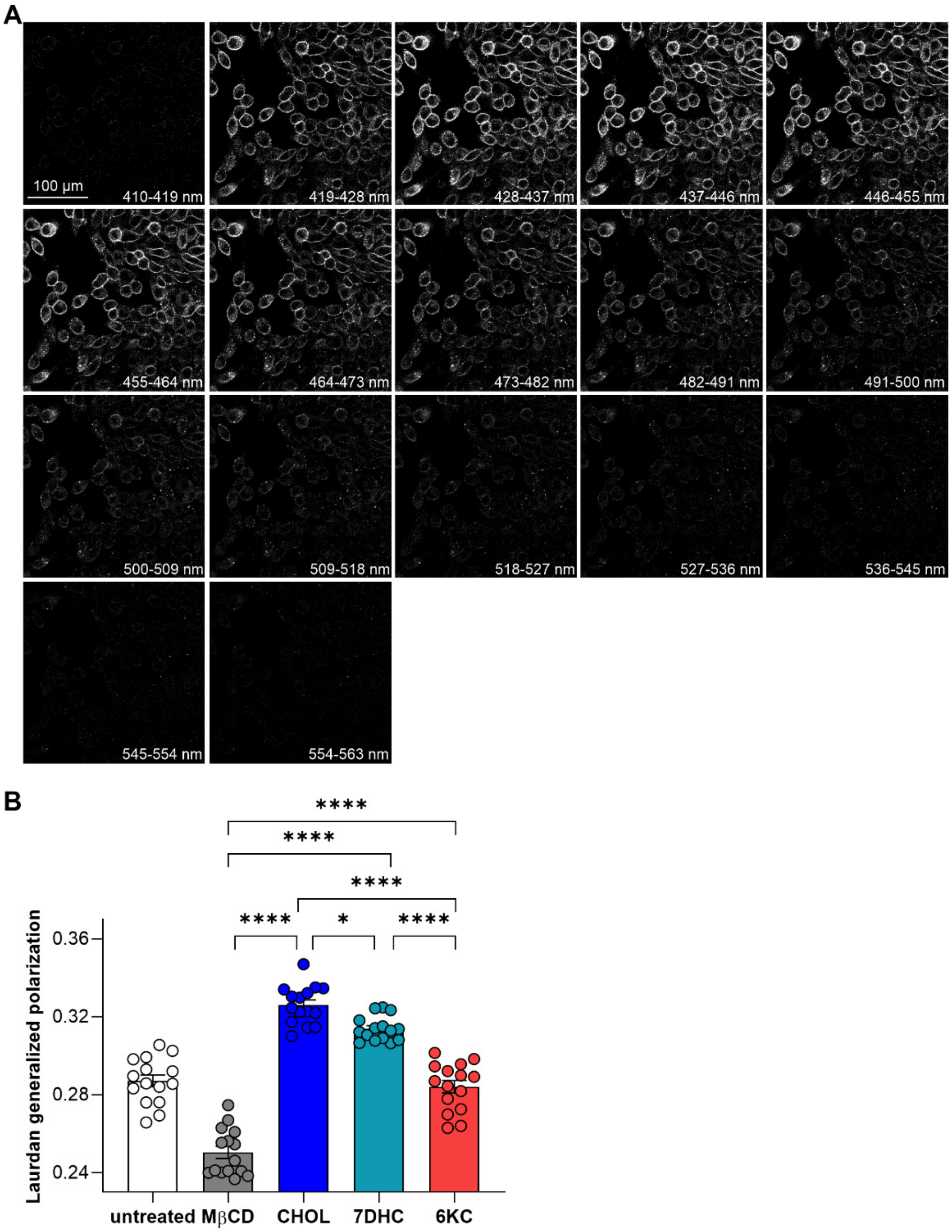

Figure S1. Examination of effects of cyclodextrin-complexed sterols on the generalized polarization of Laurdan using confocal microscopy and spectrofluorometry

(A) CHO cells grown onto an 8-well chambered coverglass were incubated in normal Ringer's solution or treated for 1 hour with native MβCD, cholesterol-MβCD (CHOL), 7-dehydrocholesterol-MβCD (7DHC) or 6-ketocholestanol-MβCD (6KC) complexes, and subsequently stained with Laurdan. Representative confocal spectral images acquired at the midplane of untreated cells after a 405-nm excitation show Laurdan intensities recorded in the displayed spectral intervals. (B) Trypsinized CHO cells were treated as above and subsequently

labeled with Laurdan, and the generalized polarization of the fluorophore negatively correlating with the extent of membrane hydration was determined using spectrofluorometry. Individual Laurdan generalized polarization values obtained in n = 15 independent samples containing approximately 100,000 cells, and their average values (± SEM) are plotted in the figure. Asterisks indicate significant differences between the samples (*$p < 0.05$ and ****$p < 0.0001$, ANOVA followed by Tukey's HSD test).

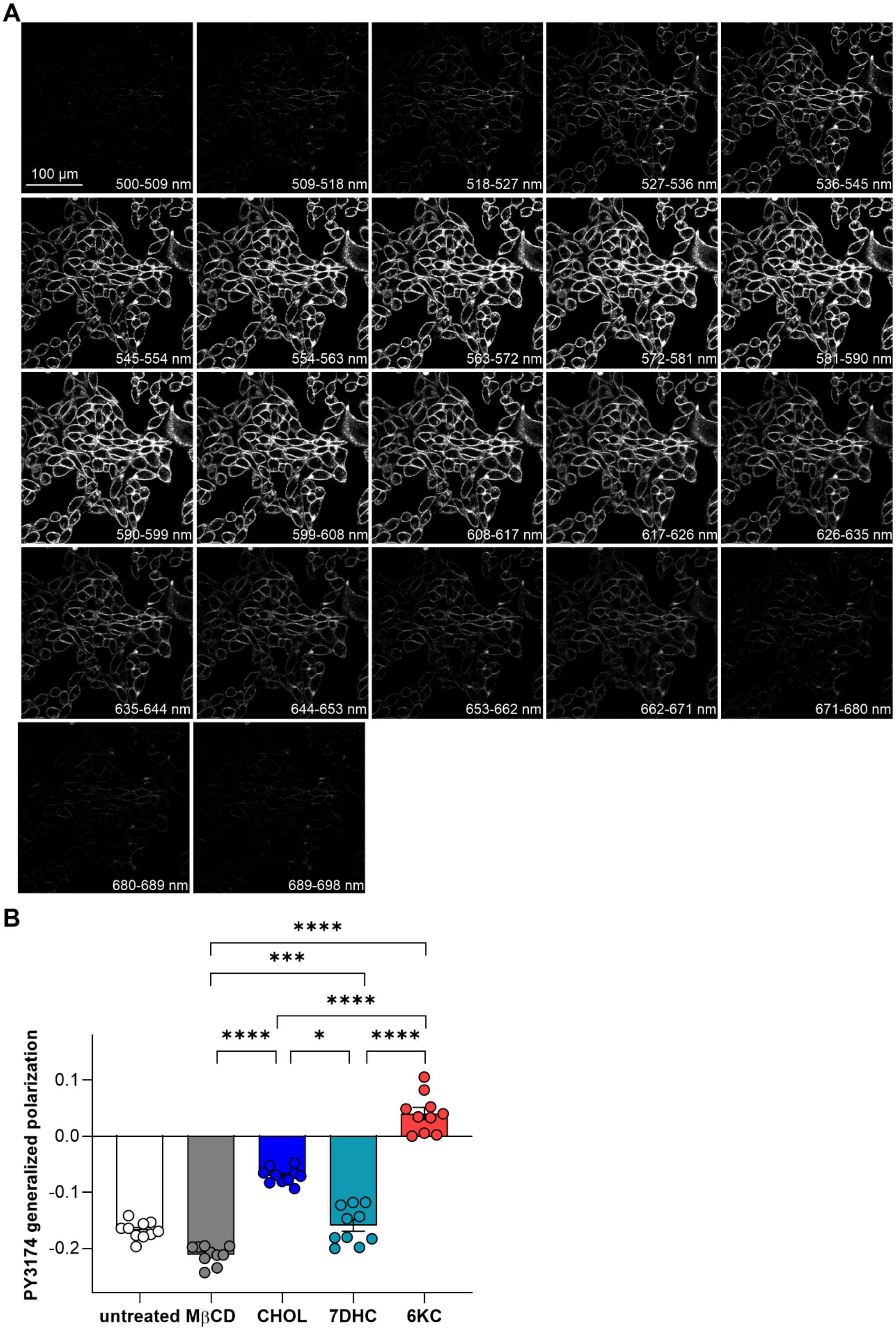
A
100 µm
500-509 nm
509-518 nm
518-527 nm
527-536 nm
536-545 nm
545-554 nm
554-563 nm
563-572 nm
572-581 nm
581-590 nm
590-599 nm
599-608 nm
608-617 nm
617-626 nm
626-635 nm
635-644 nm
644-653 nm
653-662 nm
662-671 nm
671-680 nm
680-689 nm
689-698 nm
B
PY3174 generalized polarization
0.1
0.0
-0.1
-0.2
untreated
MβCD
CHOL
7DHC
6KC
****
***
*

Figure S2. Examination of effects of cyclodextrin-complexed sterols on the generalized polarization of PY3174 using confocal microscopy and spectrofluorometry

(A) CHO cells grown onto an 8-well chambered coverglass were incubated in normal Ringer's solution or treated for 1 hour with native MβCD, cholesterol-MβCD (CHOL), 7-dehydrocholesterol-MβCD (7DHC) or 6-ketocholestanol-MβCD (6KC) complexes, and subsequently stained with PY3174. Representative confocal spectral images acquired at the midplane of untreated cells after a 488-nm excitation show PY3174 intensities recorded in the displayed spectral intervals. (B) Trypsinized CHO cells were treated as above and subsequently labeled with PY3174, and the generalized polarization of the fluorophore negatively correlating with the extent of membrane hydration was determined using spectrofluorometry. Individual PY3174 generalized polarization values obtained in $n = 10$ independent samples containing approximately 100,000 cells, and their average values (± SEM) are plotted in the figure. Asterisks indicate significant differences between the samples (*$p < 0.05$, ***$p < 0.001$ and ****$p < 0.0001$, ANOVA followed by Tukey's HSD test).

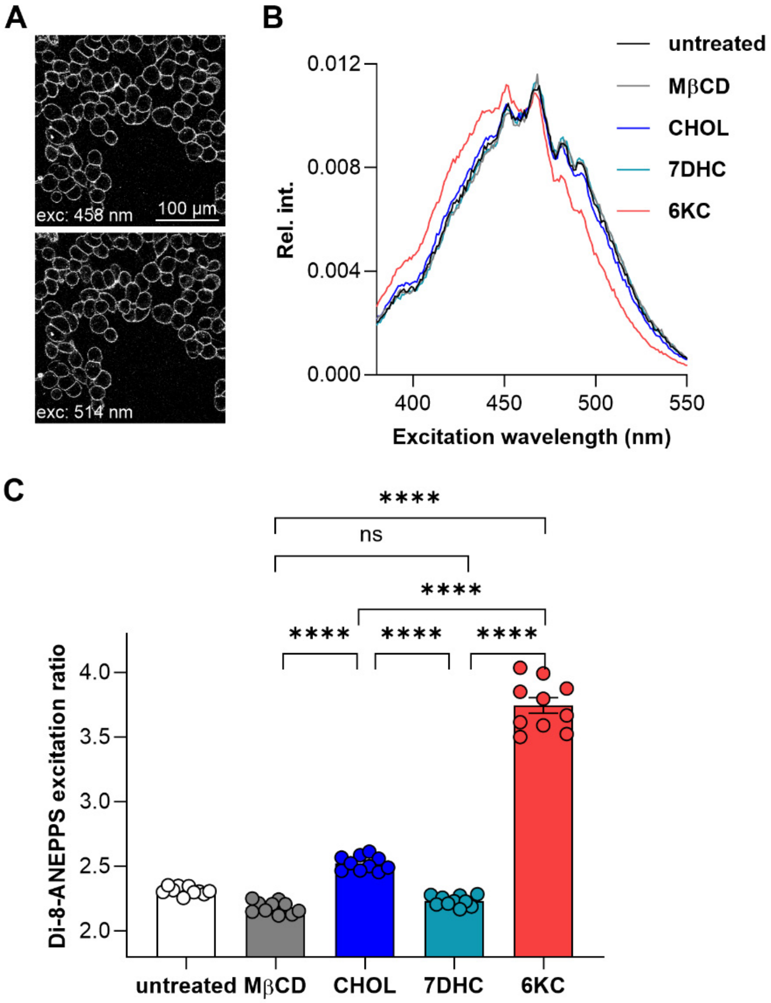

Figure S3. Examination of effects of cyclodextrin-complexed sterols on the excitation ratio of di-8-ANEPPS using confocal microscopy and spectrofluorometry

(A) CHO cells grown onto an 8-well chambered coverglass were incubated in normal Ringer's solution or treated for 1 hour with native MβCD, cholesterol-MβCD (CHOL), 7-dehydrocholesterol-MβCD (7DHC) or 6-ketocholestanol-MβCD (6KC) complexes, and subsequently stained with di-8-ANEPPS. Representative confocal microscopic images taken at the midplane of cells show di-8-ANEPPS intensities measured after excitation at 458 and 514 nm. (B) Trypsinized CHO cells were treated as above and subsequently labeled with di-8-ANEPPS. The excitation spectrum of di-8-ANEPPS was recorded with a spectrofluorometer by varying the excitation wavelength between 380 and 550 nm with an increment of 1 nm, and measuring the emission at 660 nm using slits adjusted to 5 nm on both the excitation and emission sides. Representative excitation spectra of di-8-ANEPPS display differences between untreated and treated samples. (C) Individual di-8-ANEPPS excitation ratio values positively correlating with the magnitude of dipole potential and calculated from intensities after 458-nm and 514-nm excitation in n = 10 independent samples containing approximately 100,000 cells, and their average values (± SEM) are plotted in the figure. Asterisks indicate significant differences between the samples (****$p < 0.0001$, ANOVA followed by Tukey's HSD test).

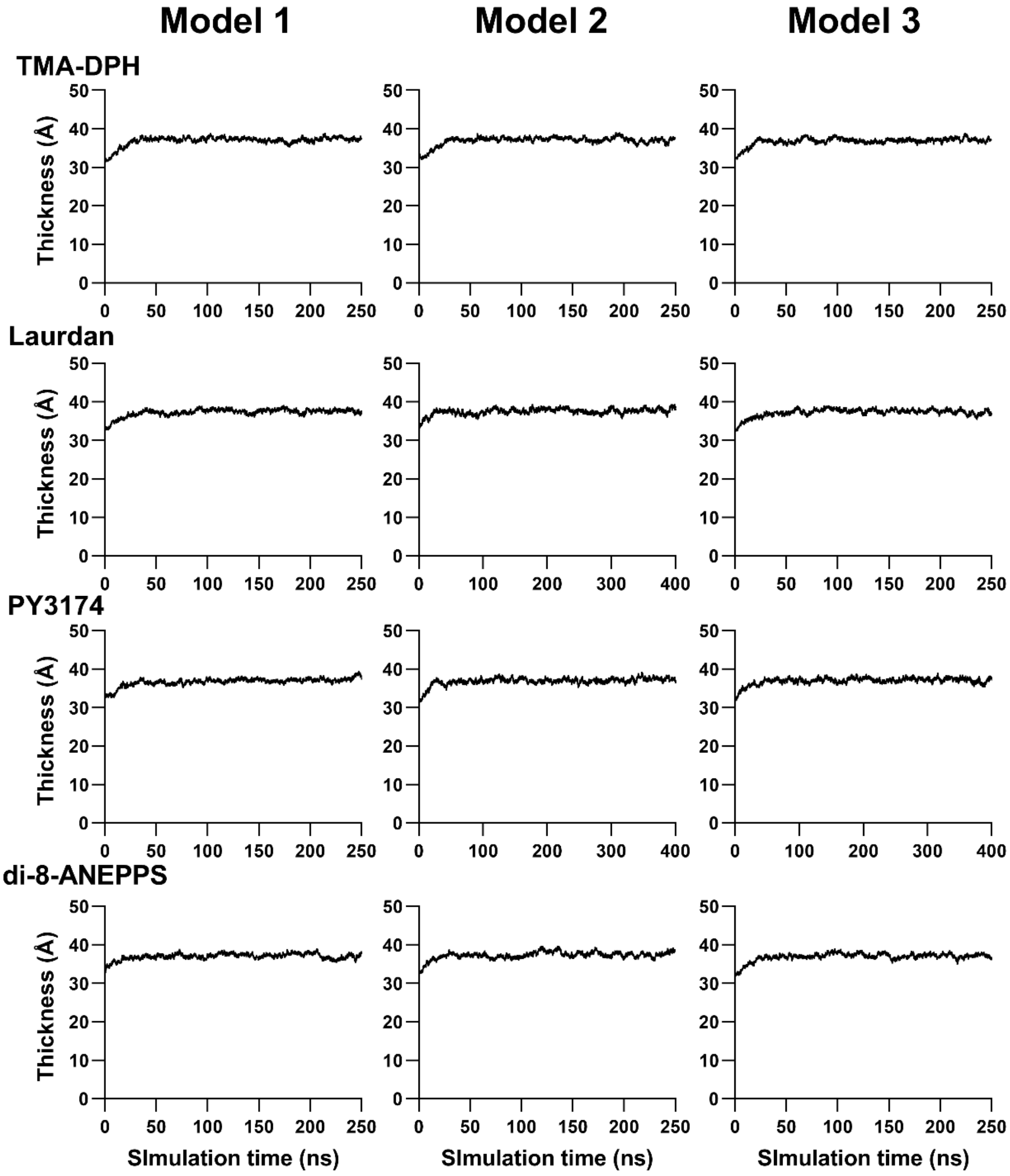

Figure S4. Time-dependent changes in the bilayer thickness during the MD simulations
TMA-DPH, Laurdan, PY3174 and di-8-ANEPPS were embedded into a pre-equilibrated lipid bilayer composed of 1-palmitoyl-2-oleoyl-sn-glycero-3-phosphocholine (POPC) at three different depths (Model 1, Model 2 and Model 3) and, after energy minimization and equilibration, production MD simulations were run for 250-400 ns. Membrane thickness values measured between the P atoms in the upper and lower layers for each system were plotted as a function of simulation time. The lack of notable changes in thickness values after the initial 30-50 ns demonstrate the overall stability of the systems.

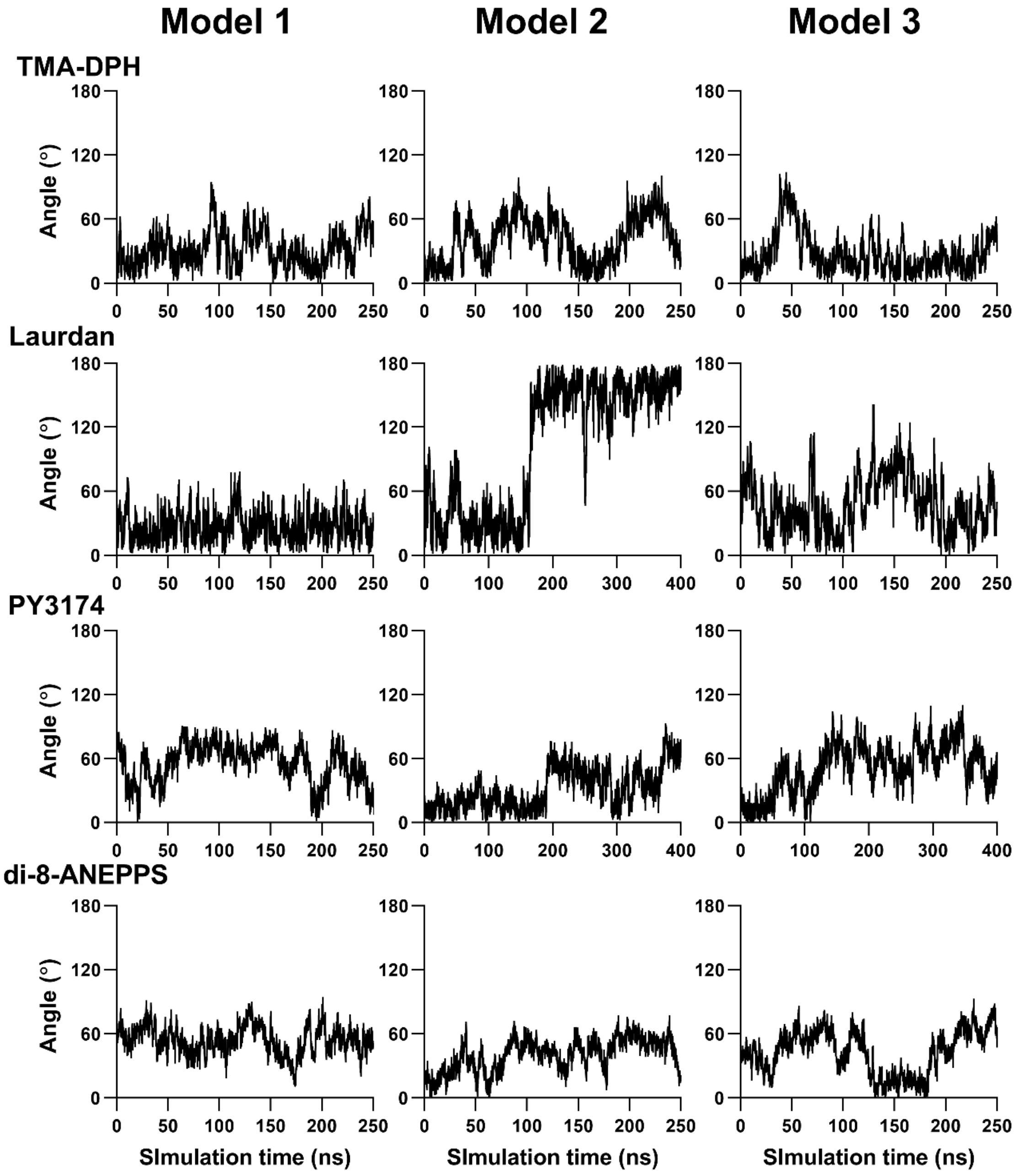

Figure S5. Time-dependent changes of dye orientation during the MD simulations

TMA-DPH, Laurdan, PY3174 and di-8-ANEPPS were embedded into a pre-equilibrated lipid bilayer composed of 1-palmitoyl-2-oleoyl-sn-glycero-3-phosphocholine (POPC) with the initial structures of fluorophores having a 30° angle to the membrane normal at three different depths (Model 1, Model 2 and Model 3) and, after energy minimization and equilibration, production MD simulations were run for 250-400 ns. Tilt angles to membrane normal were measured based on the selected atoms in each fluorophore molecule (N1 and C4 in TMA-DPH; N1 and C12 in Laurdan; N2 and N3 in PY3174; and N1 and N2 in di-8-ANEPPS) and plotted as a function of simulation time.

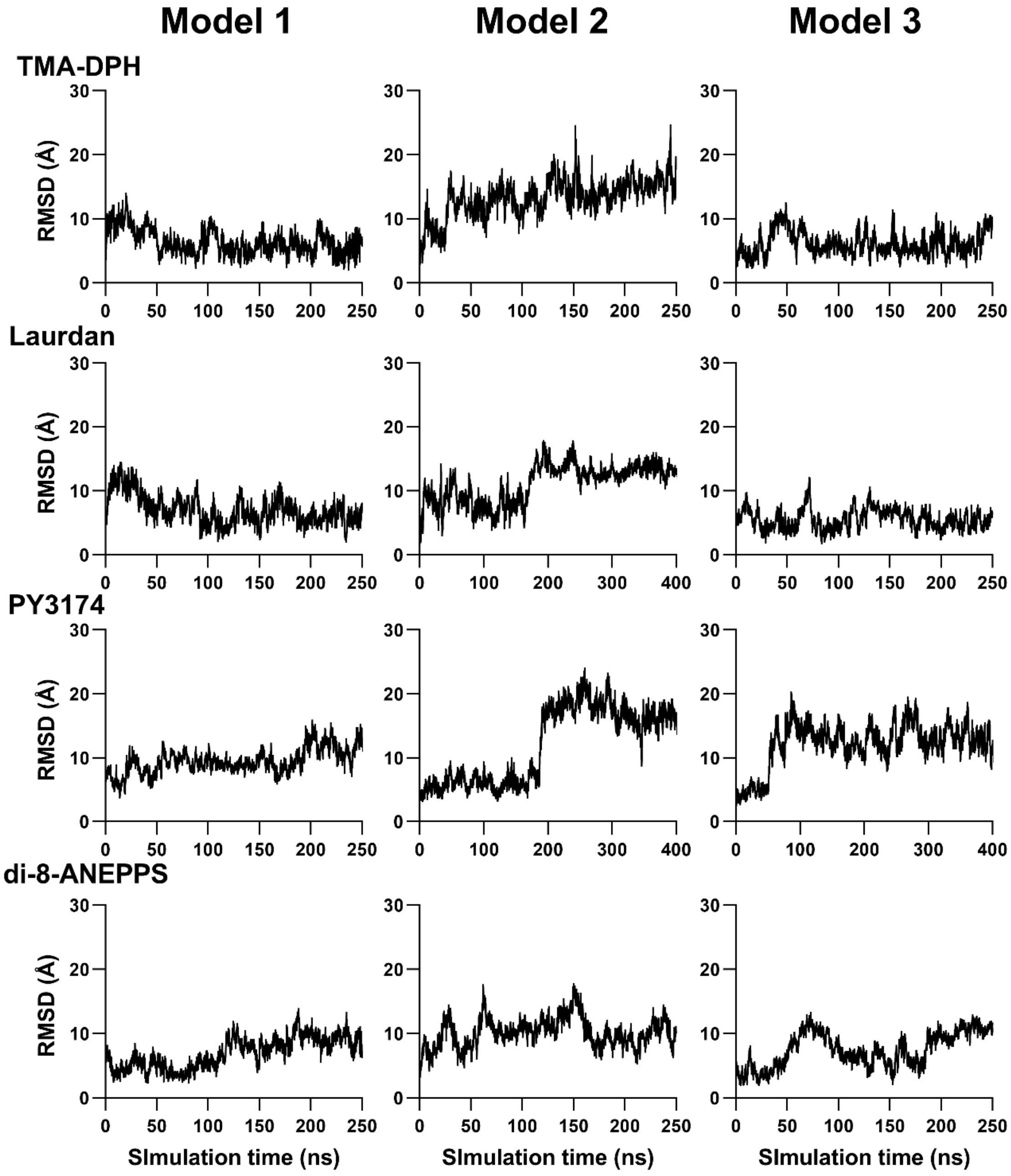

Figure S6. Time-dependent changes in the positional variation of dye heavy atoms during the MD simulations

TMA-DPH, Laurdan, PY3174 and di-8-ANEPPS were embedded into a pre-equilibrated lipid bilayer composed of 1-palmitoyl-2-oleoyl-sn-glycero-3-phosphocholine (POPC) with the initial structures of fluorophores having a 30° angle to the membrane normal at three different depths (Model 1, Model 2 and Model 3) and, after energy minimization and equilibration, production MD simulations were run for 250-400 ns. Positional RMSDs for each fluorophore molecule were calculated based on their heavy atoms and plotted as a function of simulation time during the course of the MD simulation.
